# Models of Cell Processes are Far from the Edge of Chaos

**DOI:** 10.1103/prxlife.1.023009

**Published:** 2023-12-15

**Authors:** Kyu Hyong Park, Felipe Xavier Costa, Luis M. Rocha, Réka Albert, Jordan C. Rozum

**Affiliations:** 1 Department of Physics, The Pennsylvania State University, University Park, Pennsylvania 16802, USA; 2 Department of Systems Science and Industrial Engineering, Binghamton University (SUNY), Binghamton, New York 13902, USA; 3 Department of Physics, University at Albany (SUNY), Albany, New York 12222, USA; 4 Instituto Gulbenkian de Ciência, 2780-156 Oeiras, Portugal; 5 Department of Biology, The Pennsylvania State University, University Park, Pennsylvania 16802, USA

## Abstract

Complex living systems are thought to exist at the “edge of chaos” separating the ordered dynamics of robust function from the disordered dynamics of rapid environmental adaptation. Here, a deeper inspection of 72 experimentally supported discrete dynamical models of cell processes reveals previously unobserved order on long time scales, suggesting greater rigidity in these systems than was previously conjectured. We find that propagation of internal perturbations is transient in most cases, and that even when large perturbation cascades persist, their phenotypic effects are often minimal. Moreover, we find evidence that stochasticity and desynchronization can lead to increased recovery from regulatory perturbation cascades. Our analysis relies on new measures that quantify the tendency of perturbations to spread through a discrete dynamical system. Computing these measures was not feasible using current methodology; thus, we developed a multipurpose CUDA-based simulation tool, which we have made available as the open-source Python library cubewalkers. Based on novel measures and simulations, our results suggest that—contrary to current theory—cell processes are ordered and far from the edge of chaos.

## I. INTRODUCTION

“The edge of chaos,” a term coined by Packard in 1988 [1], refers to the tendency of adaptive systems to evolve toward a dynamical regime that lies between order and disorder. In systems biology, this is often referred to as the criticality hypothesis [2], and it is closely related to work by Kauffman [3,4] and Derrida [5,6], who demonstrated that simple tunable models of gene regulation exhibit an order-to-chaos phase transition. Near this transition, it is conjectured, living systems optimally balance the rigidity required to function in a noisy environment with the flexibility required to undergo developmental, metabolic, and evolutionary processes that depend on cellular context. Dynamically, the boundary between order and disorder is often understood through the lens of trajectory separation; here, we seek to understand it through the lens of phenotypic fragility and its inverse counterpart, robustness.

The fragility of a cellular phenotype describes how easily it transitions to a different phenotype, and determines, for example, a cell’s ability to differentiate, its susceptibility to oncogenesis, and the fidelity of its signal processing. This has been measured experimentally by genetically or pharmacologically perturbing genes and measuring the impact on cellular phenotypes [7–9]. In the context of dynamical models of biomolecular networks governing cell processes, the traditional approach to understanding phenotypic fragility is inspired by the analysis of random Boolean networks (RBNs), and it considers the propagation of a large, temporary disruption to an individual component of the system (e.g., the depletion of a protein) [4]. In other words, an initial perturbation, on average, decays to extinction in the long-term dynamics of ordered (robust) systems, but it grows and spreads globally in the disordered (fragile) case.

In RBNs, the average short-term propagation of initial perturbations, as measured by the Derrida coefficient, is sufficient to determine the average long-term spreading behavior [5,6]. The Derrida coefficient measures one aspect of a defining feature of chaos: extreme dependence on initial conditions. It is closely related to the sensitivity of a Boolean network [10], and its logarithm can be interpreted as a discrete analog of the Lyapunov exponent [11]. For infinite-size Kauffman RBNs there is a rigorous connection between the Derrida coefficient and the long-term trajectory separation, which serves as an order parameter [11]. The Derrida coefficient thus indicates the critical boundary between the ordered and disordered dynamical regimes in RBNs, which occurs when its value is 1 [10].

Nonrandom, experimentally supported Boolean networks are popular tools for modeling biomolecular functional modules (regulatory mechanisms and pathways governing specific cell processes) [12,13]. As more of these models are constructed, one can ask whether an ensemble of such models exhibits properties similar to those of RBNs. In fact, many do have Derrida coefficients near 1 [14–16]. This observation lends support to the criticality hypothesis, but some caution is required; in the context of finite (and especially nonrandom) Boolean networks, the terms “order” and “chaos” are somewhat ill-defined. Unfortunately, there is no universally agreed-upon definition for these terms that is fully agnostic to the modeling framework (e.g., that applies equally to deterministic ODEs and to stochastically updated Boolean networks). Traditionally, the Derrida coefficient has been used to distinguish the ordered and chaotic regimes in the context of both thermodynamic RBNs and experimentally supported finite Boolean networks [5,6,10,14,17–19]. Alas, the connection between short-term and long-term sensitivity to initial conditions in thermodynamic RBNs does not necessarily generalize to finite systems or to ensembles of nonrandom systems. Thus, one must assess the Derrida coefficient’s ability to describe whether a finite, nonrandom (and possibly externally driven or stochastic) system exhibits characteristics typical of chaotic systems. Chief among these characteristics is sensitivity to initial conditions on long time scales. With this in mind, we consider that a finite nonrandom Boolean network is more ordered if its long-term behavior is less sensitive to initial conditions, and more chaotic or disordered if a perturbation to initial states shows long-term growth on average.

In this work, we challenge the assertion that existing nonrandom Boolean models cluster on the boundary between order and disorder by using biologically grounded measures of phenotypic fragility. Our analysis of these models reveals highly ordered perturbation responses that are obfuscated in the usual approach based on the Derrida coefficient and trajectory separation. We show that the criticality hypothesis is not valid in a battery of experimentally supported models of biomolecular networks, which represent the state-of-the-art in causal modeling in systems biology (see below). Because these networks model subsystems of whole organisms studied in isolation, our results suggest that for the criticality hypothesis to be true, criticality of living systems must arise as a mesoscale phenomenon, through the coupling of (ordered) functional modules.

Our testbed for this study is a curated collection of 72 experimentally supported, peer-reviewed Boolean network models of biomolecular functional modules found in the Cell Collective database [13], which represents the independent efforts of dozens of research groups. In all of these models, each included regulatory interaction is tagged with an experimental justification from the systems biology literature. Each node in these Boolean networks corresponds to a specific biomolecular entity (e.g., gene, protein, or cellular subprocess). These nodes each have two possible states at any given time step, which represent the activity or inactivity of the corresponding entity (e.g., transcription of a gene, phosphorylation of a protein, or initiation of a cellular process). The states of the nodes are governed by Boolean update functions, which convert the states of a node’s regulators into a binary output. Time is usually modeled as an implicit variable in these systems, and there are various methods for scheduling the update of variables. Though the steady states of the network are independent of update scheme, the oscillatory behavior of the system is not [20–22].

Indeed, the update scheme has a dramatic impact on the long-term dynamics of random networks along the order-to-chaos critical boundary [23–25]. In nonrandom models, however, rich dynamical behaviors can persist across update schemes, as illustrated in [26], though to our knowledge this has not previously been studied systematically. By thoroughly examining the impact of the update scheme on experimentally supported models, we characterize their response to perturbations in the timing and synchronization of regulatory events to explore population-level order and robustness in these systems.

In this work, we consider two extreme (and quite common) schemes: synchronous update and asynchronous update. These schemes have various tradeoffs, and either can be valid or invalid depending on modeling context. In the synchronous update, every node updates its state every time step. In other words, the state of each node at time *t* + 1 is determined by the state of its regulators at time *t*. This scheme produces fully deterministic dynamics. Due to various analytical and computational conveniences, synchronous update is a popular scheme for very large random models. Synchronous update treats all biomolecular events (e.g., gene transcription) as simultaneous, which can sometimes lead to spurious oscillations. A common approach to removing these oscillations is to consider asynchronous update schemes, though this risks destroying meaningful oscillations as well. Here, we consider a stochastic, asynchronous update scheme in which a single variable is randomly selected (uniformly) at each time step to be updated. This random selection introduces stochasticity into the dynamics and destabilizes delay-sensitive oscillations [21,22]. Thus, the asynchronous update can be viewed as a kind of timing perturbation introduced to the synchronous update.

We also take special care in handling the effect of source nodes, which usually codify a cellular context or signals external to the model. Though such nodes are common in the modeling literature, we demonstrate that they are statistically rare in random models. Moreover, we show that source nodes have a large impact on various measures of order in Boolean networks. From a dynamical perspective, a “temporary” perturbation to a source node is unique in that it will always become permanent; this stands in contrast to the behavior of constant nodes, which recover immediately after perturbation and are common in both random and experimentally derived models. In many biological applications, a perturbation to a source node is fundamentally different from a perturbation within the core of the network because source nodes often summarize the collective activity of many external components.

We consider various measures of short-term and long-term perturbation spread in both synchronous and asynchronous update schemes and in the context of fixed or perturbable source nodes using simulations. Previous work has focused on the use of short-term perturbation dynamics and statistical arguments as an avenue to estimate long-term dynamics in large networks because of the immense computational burden of ensuring that long-term perturbation measurements converge [5,17,27,28]. To meet this challenge and directly measure long-term perturbation growth in nonrandom models, we developed cubewalkers, a highly parallel GPU-based simulation toolkit, allowing us to quickly simulate many thousands of trajectories in a network simultaneously. Our software innovations, combined with the dramatic improvements in computational power over the past several decades, enable high-fidelity measurements of long-term perturbation dynamics in real-world Boolean networks with hundreds of nodes or more. These measurements are fundamental to demonstrating the true dynamical regime of experimentally supported biomolecular networks.

## II. METHODS

### A. Boolean network dynamics at the individual and population level

Boolean networks describe the regulatory dynamics of each node X by specifying its value following update, $X^{⋆}$, according to a Boolean update function $F_{X}\;:\;\{0, 1{\}}^{N}\;\to \;\{0, 1\}$. In this work, we apply a common abuse of notation in which the form of $F_{X}$ is expressed via $X^{⋆}$, because the subscript notation becomes cumbersome with long, biologically informative variable names. We define two special types of node that have unique effects on the dynamics: constant nodes, which have update functions of the form $X^{⋆}=0$ or $X^{⋆}=1$, and source nodes, which have update functions of the form $X^{⋆}=X$. More generally, update functions utilize the logical operations “AND,” “OR,” and “NOT,” which we denote by ∧,∨, and ¬, respectively. Each Boolean system with N nodes induces a state transition graph whose $2^{N}$ nodes represent all possible system states and whose directed edges indicate that the parent state can be updated in one time step to attain the child (successor) state. The attractors of a Boolean system are the terminal strongly connected components of the state transition graph (i.e., they have no edges that exit the component). Point attractors (also called steady states) consist of a single state, and oscillatory attractors (also called complex attractors) contain more than one state. The simplest type of oscillatory attractor is a limit cycle, in which the system revisits states in a deterministic order. The states that can reach an attractor via edges or paths in the state transition graph make up the basin of attraction of the attractor. In each network, the set of possible attractors can strongly depend on the update scheme used. Indeed, one of the most fundamental biomolecular circuit motifs, namely mutual inhibition, exhibits such behavior. Consider two mutually inhibiting genes, A and B, described by the simple Boolean network with update functions,

(1) $$A^{⋆}=\neg B,\;\;B^{⋆}=\neg A.$$

In the asynchronous update scheme, there are only two attractors: the steady states (A,B)=(1,0) and (A,B)=(0, 1). In the synchronous update scheme, however, there is an additional oscillatory attractor that cycles between the states (A,B)=(0, 0) and (A,B)=(1, 1). Thus, the behavior of an individual instance of a model (i.e., a single cell) is highly sensitive to the timing of the node update. This example highlights, however, that the *average* behavior of many instances (i.e., the population-level behavior) can be robust to update timing even when individual instances (cells) are not. To see this, consider the average activation value of gene A (by symmetry, the same analysis applies identically to gene B). Assuming uniformly sampled initial conditions and allowing enough time for convergence into an attractor, we observe that in the asynchronous scheme, an individual cell has a 50% probability of being in the (A,B)=(1, 0) steady state and a 50% probability of being in the (A,B)=(0, 1) steady state; thus, overall, the average value of A in the ensemble is 0.5. In the case of a synchronous update, the system has a 25% probability of being in either steady state, and 50% probability of being in the oscillatory attractor. The average value of A (and also of B) in the oscillatory attractor, however, is 0.5, and thus, overall, the average value of A in the synchronous update is also 0.5, just as it is in the asynchronous case. This behavior need not hold in general. To quantify the extent to which this behavior occurs in the test models considered, we compare the converged average node values under synchronous and asynchronous update schemes, and we compute the root-mean-squared (RMS) difference between the synchronous and asynchronous average node values across all nodes of a model, which we discuss in detail in Sec. III A 2.

### B. Models considered

Throughout this work, we consider 72 models from the Cell Collective [13] and their dynamical properties. In some cases, nodes whose update functions are constants in the originally published version of a model have been reinterpreted as source nodes in the Cell Collective, or multiple source nodes have been merged. In such cases, we defer to the original publication; in most cases, this results in replacing the update functions for several source nodes with constant-value update functions. In addition, we correct a few typographical errors in the models, remove isolated nodes, and enforce constraints that were not previously enforced when multiple nodes encode more than two values of a single entity (e.g., low, medium, or high concentration of a protein). In all, 18 models are affected in some way. We use these modified versions of the models here in an attempt to more accurately capture the biology represented in these models. Overall, we observe very little difference in the distributions of the measures considered when compared to the unaltered Cell Collective ensemble, though for some measures, the differences in individual models can be large for measures that emphasize the role of source nodes (comparisons provided in Fig. 13 in Appendix F).

**FIG. 1.**
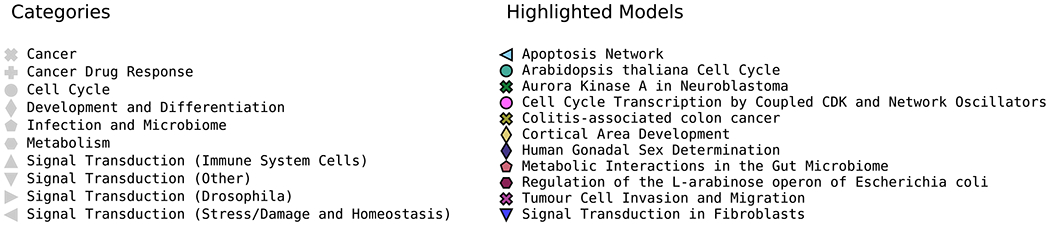
Legend indicating model categories (marker shape) and specific highlighted models (marker color).

We also highlight several models with particularly interesting dynamical features. Throughout this work, these highlighted models are indicated by colored symbols. The shape of the symbols in various plots (whether highlighted or not) describes the biological category of the model whose parameters are plotted. This correspondence is summarized in Fig. 1.

### C. Simulation and analysis software

To compute various dynamical measures, including those introduced here, we developed the cubewalkers Python library, a CUDA-based Boolean network simulator. It supports various update schemes (including user-specified schemes), node and edge control interventions, and probabilistic update rules. To simulate Boolean networks, cubewalkers parses Boolean update functions given either in algebraic form or as lookup tables. Parsed rules are compiled into a CUDA kernel via the Python interface cupy [29]. During simulation, cubewalkers executes this kernel on an array of state vectors, with each state vector representing the values of the nodes in a single network instance, or “walker.” Updates for the nodes of each walker are computed in parallel on the GPU for each time step according to the chosen update scheme. We obtain a speed-up of up to approximately 11 000 times compared to previous tools [30,31] (see Appendix A for benchmarks).

In most experiments, we use at least W=2500 independent simulations (walkers) to obtain an expected standard deviation in the average node values of less than 0.01. This convergence is remarkable because it reveals that average node values can be accurately calculated in large network models using a relatively small sample size. In a network with 50 nodes, for example, a sample of W=2500 initial states represents just over two trillionths of the state space, but is sufficient to calculate average node values at a given time step to within a few percent. Other measures we compute require more walkers to achieve the same desired accuracy; in the most extreme case, we used W=800000 walkers. We chose the number of time steps to simulate such that the largest per-node disagreement across four equal averaging subintervals was acceptably low for all Cell Collective models (below 0.0066 in the worst case, and significantly lower in most cases). In most cases, 55N+6000 time steps were sufficient, but three Cell Collective models required additional simulation time. Further details and numerical tests supporting the simulation parameters used are provided in Appendix B.

### D. Dynamical measures

The growth of small perturbations in Boolean networks is widely viewed as the hallmark of chaos in these systems [27]. In random models, this is often studied using the Derrida map, which relates the size of a perturbation at time $t_{0}$ to the size of a perturbation at time $t_{0}+1$. The Derrida map can be computed by sampling many pairs of initial states that differ in h variable values and evolving each pair of states using one synchronous time step. The average separation (Hamming distance) of the pairs becomes the numerical estimate for the value of the Derrida map at *h* [5,6]. In principle, the states reached after one time step might not be distributed uniformly in the state space, so the Derrida map does not necessarily predict whether small perturbations grow or shrink in the long term. In random Boolean networks in the thermodynamic limit (N→∞), however, whether the fixed point of the Derrida map is a finite fraction of the network is determined by the value of the map at h=1. This value is called the *Derrida coefficient* and is equal to the average sensitivity of the network [10,14]. Perturbations tend to spread to a finite fraction of the network only if the Derrida coefficient is greater than 1; this corresponds to the chaotic regime. When the Derrida coefficient is less than 1, the system is in the ordered regime in which perturbations tend to die out. A phase transition occurs on the critical boundary where the Derrida coefficient is equal to 1. Dynamically, the Derrida coefficient can be defined as

(2) $$\delta ={\langle \frac{1}{N}\sum_{i=0}^{N-1}{\left\|X\left(t_{f}\right)-X^{\left(\neg i\right)}\left(t_{f}\right)\right\|}_{1}\rangle }_{X\in 𝒯}.$$

In this formula, X is a time-dependent vector of node states, 𝒯 is the set of all trajectories in the system, and $\langle \cdot \rangle_{X\in 𝒯}$ denotes the average taken over all possible trajectories, where the initial conditions and update schedules are sampled uniformly. The trajectory $X^{\left(\neg i\right)}(t)$ is the trajectory that initially differs from X(t) only in position i and is updated in the same way as X(t) at every time step (this is important in stochastic update schemes). The comparison time $t_{f}$ is chosen such that N node updates are performed, and thus is equal to 1 in the synchronous update and to N in the asynchronous update. The summand ${∥X\left(t_{f}\right)-X^{\left(\neg i\right)}\left(t_{f}\right)∥}_{1}$ is the L1-norm (absolute difference summed, or, for Boolean inputs, the Hamming distance) between $X^{\left(\neg i\right)}\left(t_{f}\right)$ and $X\left(t_{f}\right)$ at time $t_{f}$.

**FIG. 2.**
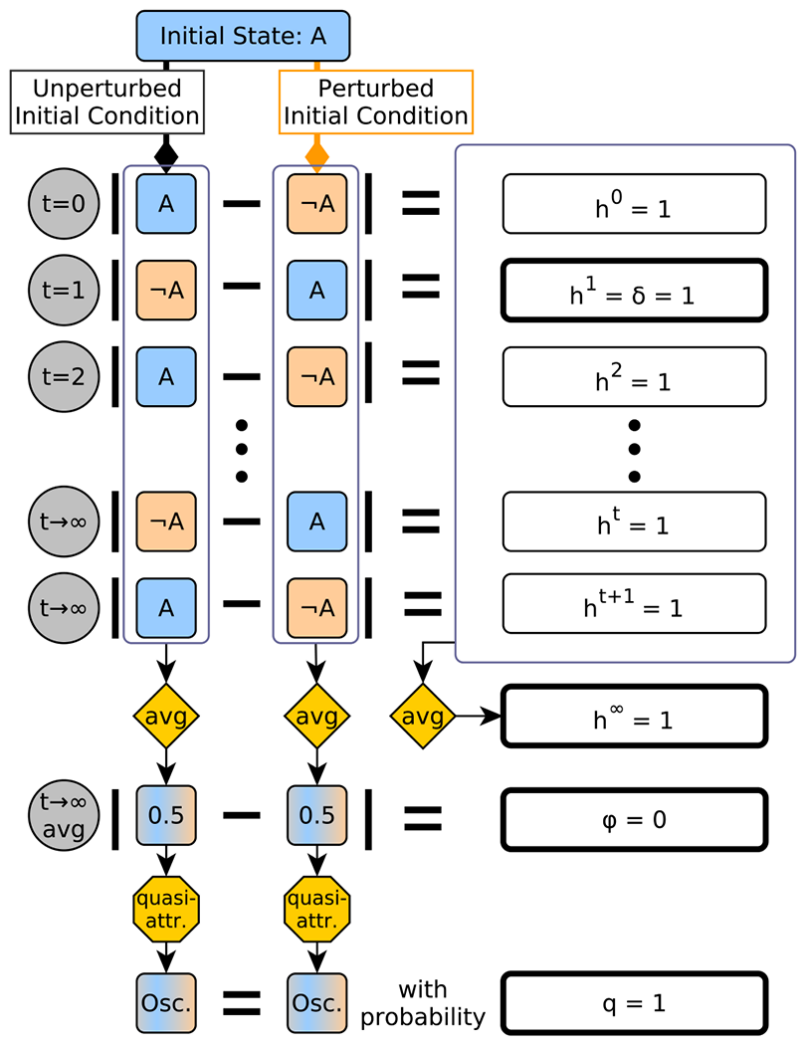
Comparison of four perturbation response measures (bold box borders) for a one-node oscillator. The unperturbed oscillator alternates between two states: its initial state A, which could be 0 or 1, and the opposite state, ¬A, which is 1 if the initial state is 0, and 0 if the initial state is 1. The perturbed trajectory begins with the oscillating node in the opposite state compared to the unperturbed trajectory, but otherwise its time evolution proceeds in the same fashion. At each time step t, the Hamming distance $h^{t}$ is computed. In the special case of $t=1,\;h^{1}$ is the Derrida coefficient δ, which evaluates to 1 in this case. Indeed, $h^{t}=1$ for all t, so the asymptotic average of the Hamming distance, which we call the final Hamming distance (denoted $h^{\infty }$) evaluates to 1 as well. Alternatively, we can compute and compare the average behavior of the two trajectories. In both cases, the node is in the 0 state for half of the time steps, and in the 1 state in the other half. Thus, the average node value is 0.5 for both trajectories, and the fragility φ, defined as the difference in these averages, is 0. Furthermore, we can consider a more coarsegrained averaging, where we compute the probability that a randomly perturbed node (in this example there is only one node to choose from) results in a different quasiattractor, i.e., a different pattern of fixed and oscillating nodes; the complement of this probability is a measure of robustness we call the quasicoherence. In this case, perturbing the initial state always results in the same quasiattractor (in which the sole node oscillates), so the quasicoherence is 1.

In addition to the Derrida coefficient, δ, we consider three other measures to describe the response of systems to small (single-node) perturbations: final (average) Hamming distance $h^{\infty }$, quasicoherence q, and fragility φ. We illustrate the intuitive meaning of these measures in the case of a single-node oscillator $A^{⋆}=\neg A$ in Fig. 2.

The *final Hamming distance* $h^{\infty }$ is a direct measure of the long-term separation between trajectories that initially differ in a single node’s value. It is defined as

(3) $$h^{\infty }={\langle \frac{1}{N}\sum_{i=0}^{N-1}{\langle {\left\|X(t)-X^{\left(\neg i\right)}(t)\right\|}_{1}\rangle }_{t\to \infty }\rangle }_{X\in 𝒯}.$$

Here, $\langle \cdot \rangle_{t\to \infty }$ indicates the average taken from any finite initial time $t=t_{0}$ to t=∞; note that the value of the time average does not depend on the value of $t_{0}$. Intuitively, $h^{\infty }$ measures the asymptotic separation (on average) between all trajectory pairs that initially differ in only one node value. Note that the Hamming distance ${∥X(t)-X^{\left(\neg i\right)}(t)∥}_{1}$ does not necessarily converge for large t (it may oscillate), necessitating the time average calculation.

The $h^{\infty }$ measure is sensitive to phase shifts; if X(t) and $X^{\left(\neg i\right)}(t)$ converge to the same limit cycle, for example, but are offset, ${∥X(t)-X^{\left(\neg i\right)}(t)∥}_{1}$ can be nonzero for all time even though the trajectories have the same long-term behavior. To distinguish this case from the case when X(t) and $X^{\left(\neg i\right)}(t)$ converge to different attractors, we propose two additional measures.

The first of these is the *fragility* φ, which we define as

(4) $$\varphi ={\langle \frac{1}{N}\sum_{i=0}^{N-1}{\left\|{\langle X(t)\rangle }_{t\to \infty }-{\langle X^{\left(\neg i\right)}(t)\rangle }_{t\to \infty }\right\|}_{1}\rangle }_{X\in 𝒯}.$$

It is expressed in the same way as $h^{\infty }$, but the time averaging occurs inside the L1-norm, rather than outside it. This removes sensitivity to phase shift, and it can be interpreted as a measure of separation in average values, rather than as an average separation. From a biological standpoint, this is desirable when a pair of trajectories with a high average separation but the same average behavior (as happens if the trajectories are time-shifted but otherwise identical) should be interpreted as phenotypically equivalent. Such trajectories may represent cells that are at different points of otherwise identical cell cycles. As a simple example, consider the system $A^{⋆}=\neg A;B^{⋆}=B$. Here, there are only two attractors in either update scheme: A will always oscillate, and B can be fixed in either value. If B is perturbed, the original and perturbed trajectories will always agree in A and differ in B, while if A is perturbed, the opposite is true and the system simplifies to the example of Fig. 2. This conclusion holds in both synchronous and asynchronous update schemes because, in the latter, we constrain the selection of the update node to always be the same in both trajectories. Thus, $h^{\infty }=1$ for this system in both update schemes. In the case when A is perturbed, however, the average value of A does not differ between the two trajectories, and thus, as in the case of Fig. 2, this perturbation contributes 0 to φ. As perturbations to B do alter the average value of B, they contribute 1 to φ and we therefore find φ=0.5 in this system overall. This indicates that half of the long-term trajectory separation due to single-node perturbations stems from time-lag effects, which are not necessarily biologically relevant. Some caution is required in this interpretation, however, as it is possible that two distinct attractors may have the same average behavior at the node level. We note that such differences would likely be extremely difficult to distinguish in a laboratory setting, and we do not observe any such attractor pairs in the networks studied here.

Another measure that can distinguish phenotypic differences from phase shifts is the *quasicoherence* q, which is closely related to the coherence measure introduced in [32]. Coherence is defined as the fraction of $\left(X(t),X^{\left(\neg i\right)}(t)\right)$ pairs that converge to the same attractor; in [32], coherence was defined only for synchronous update, but the extension to the asynchronous case is trivial. The primary barrier to adopting coherence as a measure is that attractor identification can be computationally expensive, sometimes prohibitively so. We therefore define and adopt quasicoherence as an alternative, which is defined as the fraction of $\left(X(t),X^{\left(\neg i\right)}(t)\right)$ pairs that converge to the same quasiattractor. Slightly modifying the convention of [33], we define a quasiattractor to be a pattern of fixed-node values and oscillating nodes exhibited by an attractor. Two (or more) attractors may correspond to the same quasiattractor if they share the same set of active nodes, the same set of inactive nodes, and the same set of oscillating nodes. As a simple example, consider $A^{⋆}=B;B^{⋆}=C;C^{⋆}=A$. In the synchronous update, this system has four attractors: {000},{111},{001,010,100}, and {110,101,011}. In contrast, there are only three quasiattractors: 000,111,and⋆⋆⋆, where ⋆ denotes that the node oscillates in all attractors that correspond to the quasiattractor. The quasicoherence can be written as

(5) $$q={\langle \frac{1}{N}\sum_{i=0}^{N-1}Q\left({\langle X(t)\rangle }_{t\to \infty },{\langle X^{\left(\neg i\right)}(t)\rangle }_{t\to \infty }\right)\rangle }_{X\in 𝒯},$$

where $Q\;:\;[0, 1{]}^{N}\;\times \;[0, 1{]}^{N}\;\to \;\{0, 1\}$ is defined such that Q(X,Y) is 1 if for all indices i, it holds that $X_{i}=1\;⟺\;Y_{i}=1$ and $X_{i}=0\;⟺\;Y_{i}=0$; otherwise Q(X,Y) is zero. The quasicoherence is 1 if all perturbed trajectories converge to the same quasiattractor as their unperturbed counterparts, and it is 0 if an initial perturbation to a single node always results in a different quasiattractor.

The quasicoherence, unlike the final Hamming distance and fragility, does not distinguish between the case when trajectories converge to very similar (but not equal) steady states from the case when they converge to very different steady states. Because the time averaging is conducted before comparison, it is not sensitive to phase shifts either. The quasicoherence is useful when long-term changes in the expression of even a small number of genes are phenotypically important. The fragility and quasicoherence are related to each other in that the fragility can be interpreted as a rescaled “fuzzy” version of the quasicoherence, as explained in Appendix C.

We compute these dynamical measures $\left(h^{\infty },q\right.$, and $\left.\varphi \right)$ numerically for each network in the Cell Collective using a simulation-based approach. First, we sample 2500N initial states, produce a copy of each, and perturb each copy in exactly one node (for a total of 5000N initial states). Each initial state is evolved forward in time for $T=T_{b}+T_{w}$ time steps, and the various time averages are taken over the last $T_{w}$ time steps, as described in Appendix B. This is done in both the synchronous and asynchronous update schemes. The Derrida coefficient is computed using one synchronous time step or N asynchronous time steps using 100 000 initial samples (for a total of 200 000 initial states when considering the perturbation).

In addition, to probe the effect of source nodes (nodes whose update functions are of the form $A^{⋆}=A$) in Boolean networks, we consider “fixed source” versions of these five measures in which the perturbed nodes may not be source nodes and in which all instances of N in the formulas are replaced by the number of nodes that are not source nodes. Importantly, constant nodes remain perturbable in these cases, as do nodes that become fixed as a direct consequence of the source node values. All other parameters are unchanged.

Taken together, this results in four variations of each measure: two possible choices of update, indicated by a subscript s for synchronous and a for asynchronous, and two possible choices for how to treat source nodes, indicated by subscript f or p for fixed source nodes or perturbable source nodes, respectively. For example, $\varphi_{s,f}$ indicates the fragility computed using the synchronous update and not allowing for source nodes to be perturbed, while $\varphi_{a,p}$ indicates the fragility computed using the asynchronous update and allowing source nodes to be perturbed. In total, we consider 16 measures of node perturbation response. The four variants of the Derrida coefficient δ measure short-term perturbation response. The four variants of the final Hamming distance $h^{\infty }$ measure long-term perturbation response in a manner that is sensitive to phase shifts. The four variants of the fragility φ measure long-term perturbation response in a manner that is insensitive to phase shifts. Finally, the four variants of the quasicoherence q measure the probability that a node perturbation does not induce a long-term change in quasiattractor.

## III. RESULTS

### A. The effects of synchronization perturbation

We first consider the effects of perturbations to the synchrony of biomolecular events. By comparing network dynamics under synchronous and asynchronous update, we consider an extreme version of this timing perturbation in which no two node states can update simultaneously. We study this at the level of single networks (akin to studying individual cells) and at the level of network populations (akin to studying populations of cells). At the level of individual networks, we examine the effect of perturbations on the range of possible long-term behaviors, whereby a reduction of this range corresponds to increased order. At the population level, a synchronously updated network is timing robust if it retains the average population-level behavior even when the synchrony of the biomolecular events it encodes is disrupted. In other words, a Boolean network exhibits a robust and ordered response to timing perturbations at the population level if its average node values do not depend (much) on the choice of update scheme.

#### 1. Synchrony perturbation confers order by destroying attractors

The attractor repertoire of Boolean models (and specifically, the oscillatory attractors) depends on the update scheme [20,22]. In general, there are more attractors under synchronous update than under asynchronous update. As synchronous update is deterministic, its oscillatory attractors are always limit cycles. Attractors that only exist for synchronous update rely on the exact timing of updates (such that multiple nodes change state at the same time), and they disappear in the case of variations of the update timing, causing the system to have more orderly behavior [21]. We identify several models in the Cell Collective with this property and characterize the mechanisms underlying it by studying simplified models that are obtained by percolating the fixed value of source nodes, on eliminating a self-edge-free node and plugging in its update function into the function of its targets [34,35], and on merging nodes with similar regulatory roles.

In Appendix D, we discuss several models in detail, with an emphasis on the biological implications of their update scheme dependence or robustness. Three update-scheme dependent models relevant to this section are the Cell Cycle Transcription by Coupled CDK and Network Oscillators (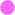) [36], Aurora Kinase A in Neuroblastoma (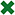) [37], and Regulation of the L-arabinose operon in *Escherichia coli* (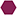) [38] models. These have attractors under synchronous update that vanish under asynchronous update. In the first two models, these attractors are biologically meaningful and arise from a delay-dependent interaction between a positive and negative feedback loop. In the third model, the additional attractors under synchronous update are biologically spurious [38] and arise from a positive feedback loop in a manner similar to the example of Eq. (1). These models illustrate that the biological interpretation of a Boolean network can depend strongly on update scheme. Timing perturbations can destabilize oscillations that depend on specific delays between events by making them stochastic. This can lead to a decrease in the range of behaviors available to individual cells, ultimately resulting in dynamics that are more constrained and orderly.

#### 2. Timing-robust order emerges in cell populations

**FIG. 3.**
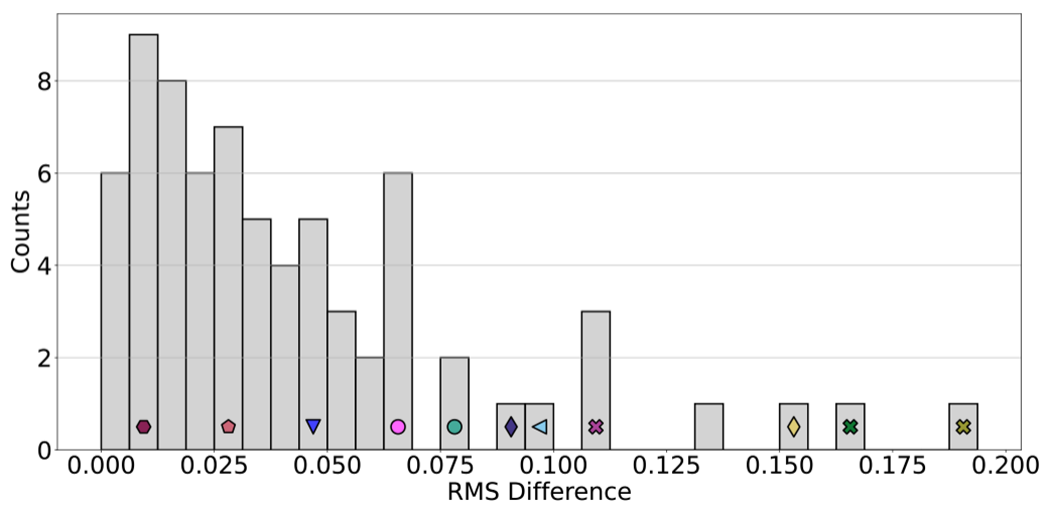
Distribution of update dependence in the Cell Collective. The root mean squared (RMS) difference between the node values when using synchronous or asynchronous update, as defined in Sec. II A, is shown. The peak near zero indicates a high degree of timing robustness in the Cell Collective models. Representative models are indicated by symbols according to Fig. 1.

Though the attractor repertoire of models can be sensitive to the update scheme at the level of individual cells, we observe that robustness to timing perturbations typically emerges at the cell population level. This suggests that populations of cells exhibit order that is not necessarily observable at the individual level. In almost all cases, the difference between the converged average node values in the synchronous and asynchronous updates is extremely small (see Fig. 3). Notable exceptions include the Colitis-associated Colon Cancer (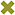), Aurora Kinase A in Neuroblastoma (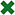), and Cortical Area Development (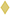) models. These three models have the three highest values of RMS difference and thus exhibit the least orderly response to timing perturbation.

Models with no difference at all between update schemes, such as the Toll Pathway of Drosophila Signaling Pathway model [39], exhibit a kind of monostability in which only a single globally stable fixed point exists for each combination of source node values, regardless of update scheme; these models are highly ordered. In some cases, a model is monostable for some, but not all, of its source node configurations; the Regulation of the L-arabinose operon in the *Escherichia coli* (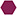) model [38] is one such example, and it illustrates that a low RMS difference is possible in models with update-dependent attractors. The model is monostable for 11 of the 12 biologically meaningful configurations of its source nodes (which encode three levels of external arabinose, the presence or absence of external glucose, and bound/unbound AraC protein). In the last combination, there are two point attractors and four update-dependent attractors. Despite this, as in the example of Eq. (1), the average node values are not affected by the additional attractors. Similarity between update schemes can also arise in more subtle ways. For example, the Metabolic Interactions in the Gut Microbiome (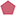) model [40] is primarily driven by a small, update-independent subnetwork. This results in an update-independent attractor that dominates the state space, with the remaining state space split between two similar attractors (see Appendix D for details).

In cases when timing robustness fails to emerge, the network typically has a large number of states that can evolve to more than one attractor in the asynchronous update. Under synchronous update, each of these states must deterministically evolve to only one attractor. When these states are heavily biased toward one attractor over another, the network can exhibit desynchronization sensitivity. The phenomenon explains the most extreme case of average node value sensitivity to update scheme that we have observed: the Colitis-associated colon cancer (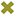) model [41]. In this case, the behavior is driven by a small three-node subnetwork that is highly update-dependent; we analyze this subnetwork in detail in Appendix D, where we also examine the update dependence of the full Cortical Area Development (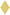) model [42], together with an improved version also presented in [42].

We caution that careful consideration of the underlying biology is always important when analyzing these models and selecting an update scheme, even when population-level average node values are fairly robust to timing perturbations. For example, the Apoptosis Network (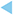) model [43] has an RMS difference in average node values that, though higher than the median, is low in absolute terms (near 0.1; see Fig. 3). Despite this, the likelihood of achieving apoptosis in this model strongly depends on update scheme: apoptosis is twice as likely under asynchronous update (see Appendix D for details).

Though cases of update scheme dependence often highlight interesting regulatory mechanisms, we emphasize that population-level desynchronization robustness is by far more common in the Cell Collective. In combination with the results of the previous section, this points to an order in the average states of nodes that is hidden when these biomolecular networks are viewed as isolated entities but that is revealed when they are viewed as members of an ensemble.

### B. The effects of transient state perturbations

In the previous section, we discussed the effects of timing perturbations in Cell Collective models; we now consider the effects of transient node perturbations in which the state of a variable is temporarily altered. We emphasize the comparison between the short-term response measured by the Derrida coefficient (δ) and long-term responses measured by the quasicoherence (q), final Hamming distance $\left(h^{\infty }\right)$, and fragility (φ), which are defined in Sec. IID and differ in how long-term changes to trajectories are quantified. We also consider the impacts of internal perturbations separately from those of environmental changes by considering two cases for all measures: perturbable and fixed source nodes, emulating a variable or static cellular context, respectively.

#### 1. The prevalence of source nodes in the models has a strong influence on trajectory separation

Previous studies did not consider the fact that the variables of Boolean network models fall into two qualitatively different categories: independent variables (represented by source nodes in the network) and variables whose values are determined by their interactions (represented by nodes with incident edges in the network). Source nodes are rare in most types of RBN ensembles. We determined (see Appendix E) that in any ensemble of finite random networks obeying widely used independence assumptions, on average more than 75% completely lack source nodes. This stands in stark contrast to the Cell Collective; only nine of the 72 models we studied are source-free, and the average number of source nodes in these networks is 4.94 (median 3, maximum 33) (see Fig. 12 in Appendix F for the full distribution). Note that these statistics and the distribution of the number of source nodes do not include constant nodes or source nodes for which only one value is ever considered in the analysis of a model’s original publication. The number of constant nodes in random models is much less tightly constrained than the number of source nodes, thus the frequency of constant nodes in our test ensemble could plausibly be obtained in random models (see Appendix E).

**FIG. 4.**
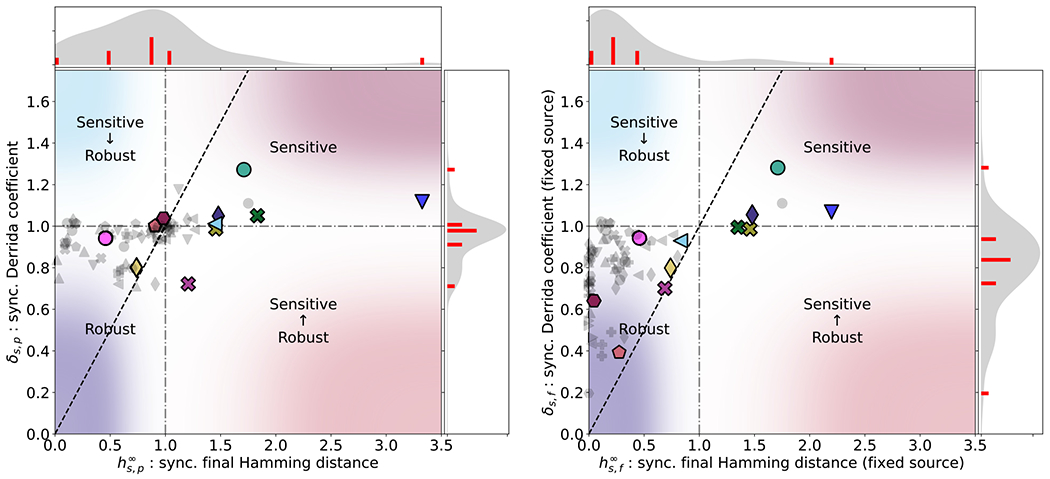
Short- and long-term perturbation responses in the Cell Collective measured in a phase-sensitive way. In the “Robust” regime (lower left quadrant) both short-term and long-term responses are below 1, which indicates perturbation extinction and is characteristic of ordered dynamics. In the “Sensitive” regime (upper right quadrant) both short-term and long-term responses are above 1. This indicates perturbation growth, which, in the extreme case, is characteristic of disordered or chaotic dynamics. The other two quadrants indicate cases of disagreement between the short-term and long-term responses. The short-term perturbation response δ has a slight correspondence with the long-term perturbation response under the specific setting when $h^{\infty }$ is monitored and synchronous update is used, in which the phase shifts are conserved. The relationship between short- and long-term responses is stronger when source nodes are fixed (right panel). The dashed line indicates the y=x diagonal. The symbols indicate the model categories and highlighted models as defined in Fig. 1.

Dynamically and biologically, source nodes play an important role. In biology parlance, they often describe the cellular *context*, or configuration of the external environment and of intracellular mechanisms outside the scope of the model under study. Often, a change to the value of a source node represents an enormous shift in this context. This is because a change in the value of a source node is not a temporary dynamical perturbation, but a permanent alteration of the modeling context. Dynamically, this is reflected in the distribution of δ and $h^{\infty }$ in the Cell Collective ensemble (see Fig. 4). When source nodes are perturbable in the synchronous update, we find that the distribution of $\delta_{s,p}$ peaks very close to 1. This corroborates previous observations[14,16] in Boolean models of biological systems. However, an abundance of source nodes tends to increase δ in these models, in some cases dramatically, because the ultimate size of a perturbation that begins at a source node is always bounded below by one (in contrast, constant nodes tend to decrease δ because they are guaranteed to recover from any perturbation). Furthermore, many Cell Collective models are concerned with how signals, represented by source nodes, are processed by cells, meaning that—by design—such models tend to be sensitive to the values of these source nodes.

By isolating the effects of source nodes on the δ, we can begin to understand the degree to which the overall perturbation response in cellular systems is governed primarily by factors internal to specific functional modules (nonsource nodes), or by the interplay between these modules and their environment (source nodes). When we restrict attention to the system’s response to internal perturbations only, we see that δ is no longer centered near 1. Rather, the distribution shifts dramatically to the “ordered” regime (below 1). For example, the Metabolic Interactions of the Gut Microbiome (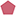) model has δ≈1 when source nodes are candidates for perturbation but only ≈0.39 when they are not. In the asynchronous case, defined in Eq. (2), δ is more tightly clustered, but overall, δ shows very little dependence on the update scheme (see Fig. 14 in Appendix F for a direct comparison). This suggests that, on short timescales, the disorder that arises from node perturbations does not couple with the noise that arises from disruptions to update synchrony.

A few models do not follow the general trend and exhibit δ higher than 1. One example is the *Arabidopsis thaliana* Cell Cycle (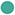) model [44], which has the highest value of δ (greater than 1.2 in both update schemes). This 14-node, source-free model has an abundance of regulators (average in-degree of 4.71), a significant percentage of which (42%) are negative regulators. The complexity of the regulation is likely the reason for the high observed initial separation of trajectories following an initial perturbation to a single node.

In the thermodynamic limit of random Boolean networks, there is a very strong relationship between δ and $h^{\infty }$. Whether or not this holds in the Cell Collective is investigated in Fig. 4. The quadrants of the two panels of Fig. 4 show whether the perturbation response indicates perturbation growth or decay in the short- or long-term (perturbation growth being a hallmark of chaos). Following [18,45], the short-term perturbation response of the models, as measured by δ, suggests ordered dynamics in the bottom two quadrants and chaotic dynamics in the top two quadrants, though we emphasize that, unlike in random models, the short-term perturbation response seen here is not necessarily predictive of the long-term response. The long-term perturbation response, as measured by $h^{\infty }$, suggests robustness (a hallmark of ordered dynamics) in the left two quadrants and sensitivity (a hallmark of chaotic dynamics) in the right two quadrants. In the Cell Collective models, we observe a slight correspondence between δ and $h^{\infty }$ under synchronous update. No correspondence of δ and $h^{\infty }$ was found for asynchronous update (see Fig. 15 in Appendix F). It is somewhat expected that the correspondence between δ and $h^{\infty }$ would be stronger in synchronous update, where phase shifts within oscillatory attractors are always persistent. In contrast, phase shifts often decay in asynchronous update. When source nodes are not perturbable, δ serves as an upper bound for $h^{\infty }$ in the robust regime, and as a lower bound for $h^{\infty }$ in the sensitive regime (see Fig. 4, right panel). For fixed source nodes, $h^{\infty }$ varies wildly when δ≈1, which is characteristic of systems near a phase boundary. Note that both δ and $h^{\infty }$ are skewed more toward the robust regime than in the traditional approach of perturbable source nodes, shown in the left panel.

When source nodes are not perturbable, $h^{\infty }$ decreases dramatically for many models (see Fig. 16 in Appendix F). This is likely due to the large number of Cell Collective models that describe how functional modules integrate and respond to external signals, leading to a bias for source nodes with significant downstream effects. For example, as previously discussed, the Regulation of the L-arabinose operon in *Escherichia coli* model (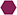) is monostable in most of its input configurations. This leads to very small $h^{\infty }$ when source nodes are not perturbable, despite the fact that this model has a slightly above-average $h^{\infty }$ when its source nodes are potential perturbation targets.

Models of functional modules with more complex internal dynamics, such as the Signal Transduction in Fibroblasts (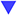) model [46], can also be greatly affected by source nodes. This model stands out in its high value of $h^{\infty }$, despite its only slightly elevated Derrida coefficient $\left(\delta_{s,p}=1.12\right)$. This 130-node model describes the response of a specific cell type to nine external signals (growth factors, cytokines, stress). The model has a very large number of oscillating attractors (hundreds for each input configuration). A key contributing factor to this rich oscillating dynamics is the large fraction (∼25%) of nodes with negative self-regulation in this model. In addition, 32 out of the 44 nonmonotonic update functions in the Cell Collective are found in this model. The signals modulate the complex internal dynamics, but do not completely control them; thus the horizontal position of this model in Fig. 4 is further to the left when source nodes are fixed (right panel), but it remains the model with the highest $h^{\infty }$.

The Tumour Cell Migration and Invasion (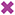) model [47] stands out in that it has a low value of δ, but a high value of $h^{\infty }$ in synchronous update when source nodes are perturbable (a similar, less extreme, pattern is observed under asynchronous update as well; see Fig. 15 in Appendix F). This model describes the processes necessary for cancer cell metastasis, including an epithelial to mesenchymal cell fate change, gain of motility, and the ability to invade the neighboring tissue (these four phenotypes are represented by nine, update-independent point attractors). The model’s two inputs describe an internal signal (DNA damage) and an external signal from the cell’s microenvironment. The nonmonotonic change in time of the Hamming distance persists in the input combination most relevant to cancer cells. One factor that contributes to a low δ (below 1) is the strong canalization of the model’s functions, which are biased heavily toward the “OFF” state. This causes many perturbed trajectories to immediately realign, resulting in a low δ. Though most trajectory pairs quickly align, those that do not tend to dramatically increase their separation, converging into very distinct attractors and resulting in a higher $h^{\infty }$.

Collectively, the Regulation of the L-arabinose operon in *Escherichia coli* model (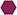), Signal Transduction in Fibroblasts (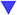), and Tumour Cell Migration and Invasion (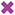) models illustrate the strong influence of source nodes in controlling the perturbation response. In the Regulation of the L-arabinose operon in *Escherichia coli* model, the dynamics are almost fully controlled by the source nodes. In the Signal Transduction in Fibroblasts (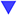) model, a great deal of dynamical freedom remains even when source nodes are frozen due to an abundance of self-inhibition and nonmonotonic regulation, but the perturbability of source nodes exaggerates these effects. In the Tumour Cell Migration and Invasion (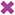) model, the perturbation of source nodes produces a pronounced pattern of initial perturbation decay followed by perturbation growth due to extreme canalization of individual regulatory elements.

#### 2. Perturbation response beyond trajectory separation

**FIG. 5.**
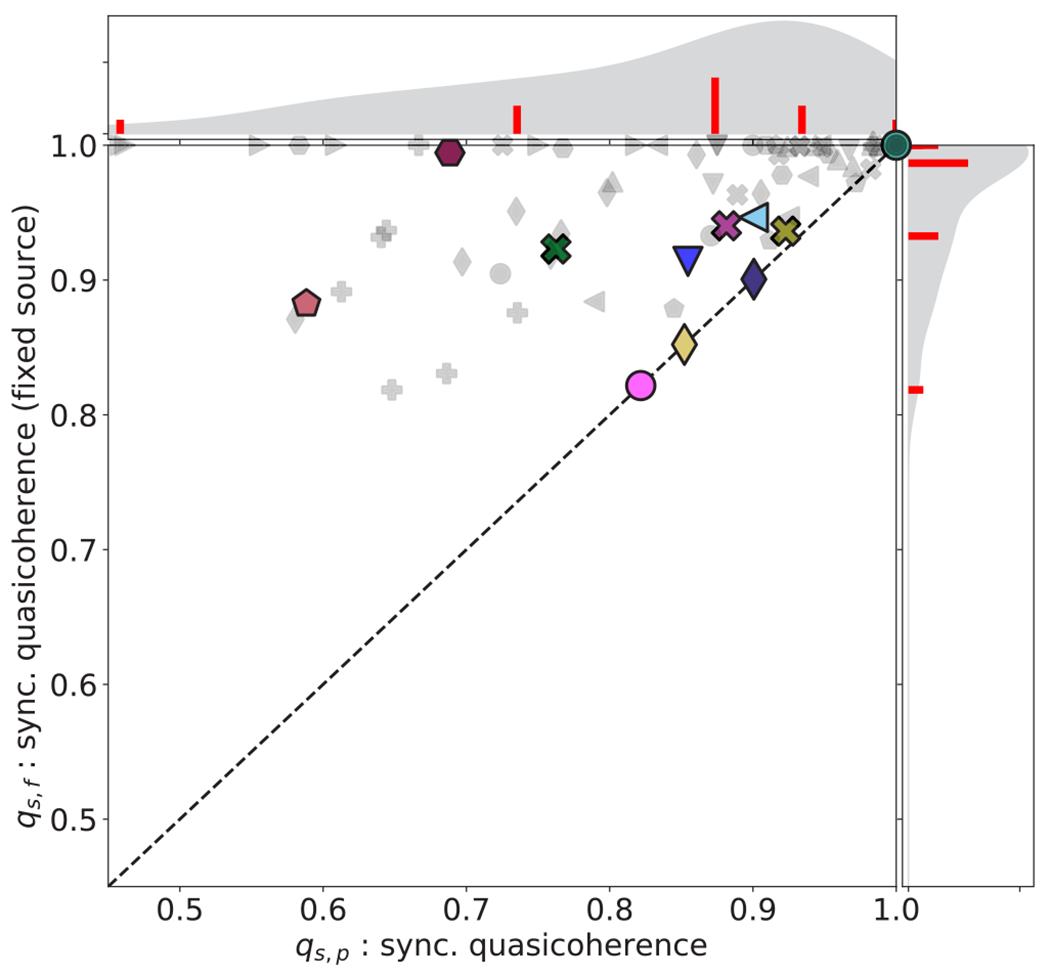
Scatterplot of the synchronous quasicoherences of the Cell Collective models when source nodes are (*x* axis) or are not (*y* axis) candidates for perturbation (the asynchronous distribution is available in Fig. 17 of Appendix F). When the values of source nodes are fixed, the quasicoherence values are tightly clustered around 1, indicating a high degree of phenotypic robustness. The symbols indicate the model categories and highlighted models as defined in Fig. 1.

**FIG. 6.**
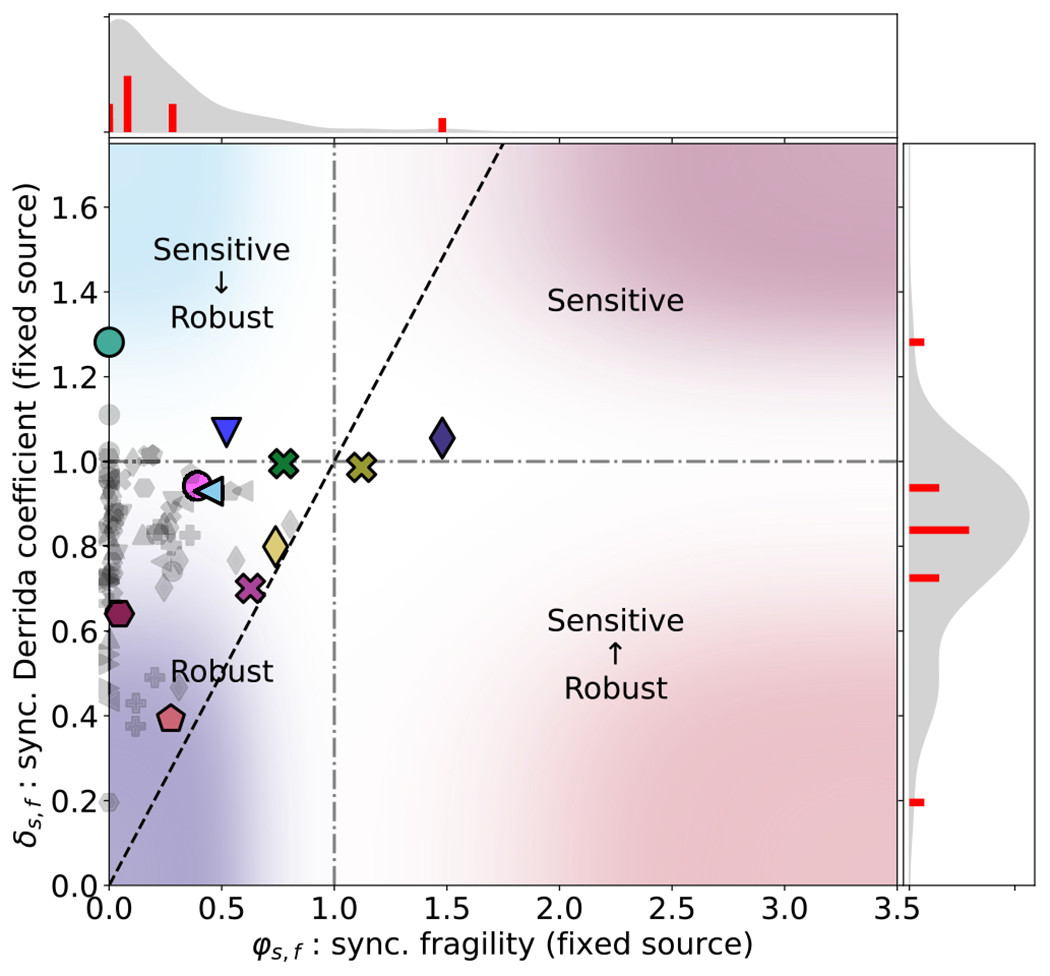
Short- and long-term perturbation responses in the Cell Collective measured in a phase-insensitive way. In the “Robust” regime (lower left quadrant), both short-term and long-term responses are below 1, which indicates perturbation extinction and is characteristic of ordered dynamics. In the “Sensitive” regime (upper right quadrant), both short-term and long-term responses are above 1. This indicates perturbation growth, which, in the extreme case, is characteristic of disordered or chaotic dynamics. The other two quadrants indicate cases of disagreement between the short-term and long-term responses. In contrast with the traditional approach depicted in the left panel of Fig. 4, this figure illustrates perturbation response when source nodes and phase shifts are accounted for. Most models show a substantially more robust perturbation response when these factors are taken into consideration. The symbols indicate the model categories, and highlighted models as defined in Fig. 1.

**FIG. 12.**
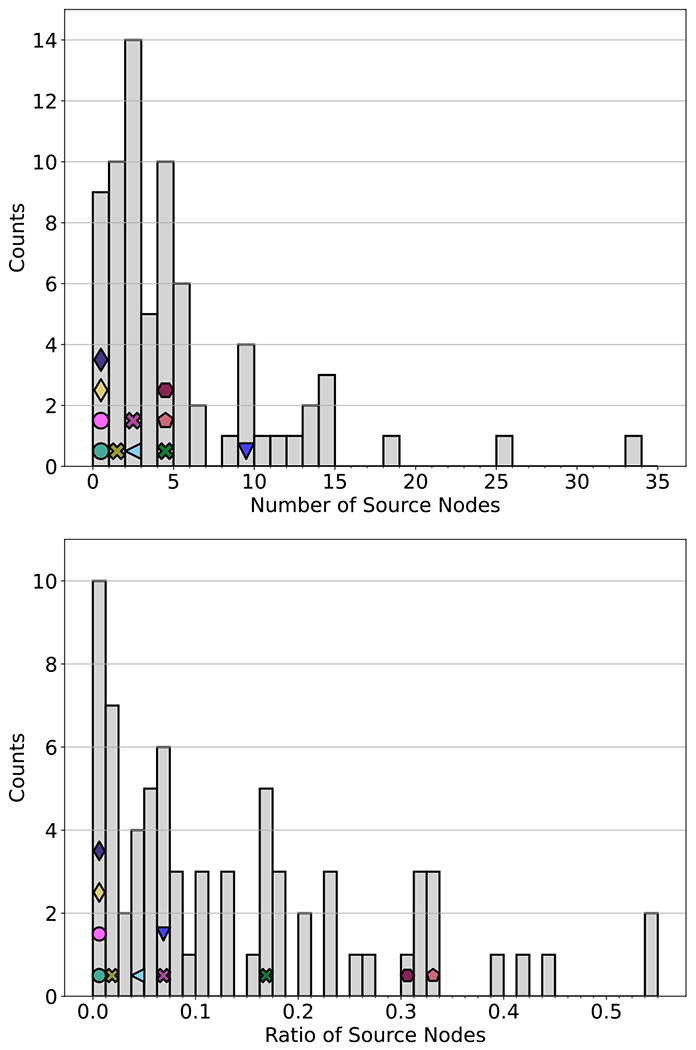
The distribution of the Cell Collective models based on the number of source nodes (top) and the ratio of the number of source nodes to the total number of nodes (bottom).

**FIG. 13.**
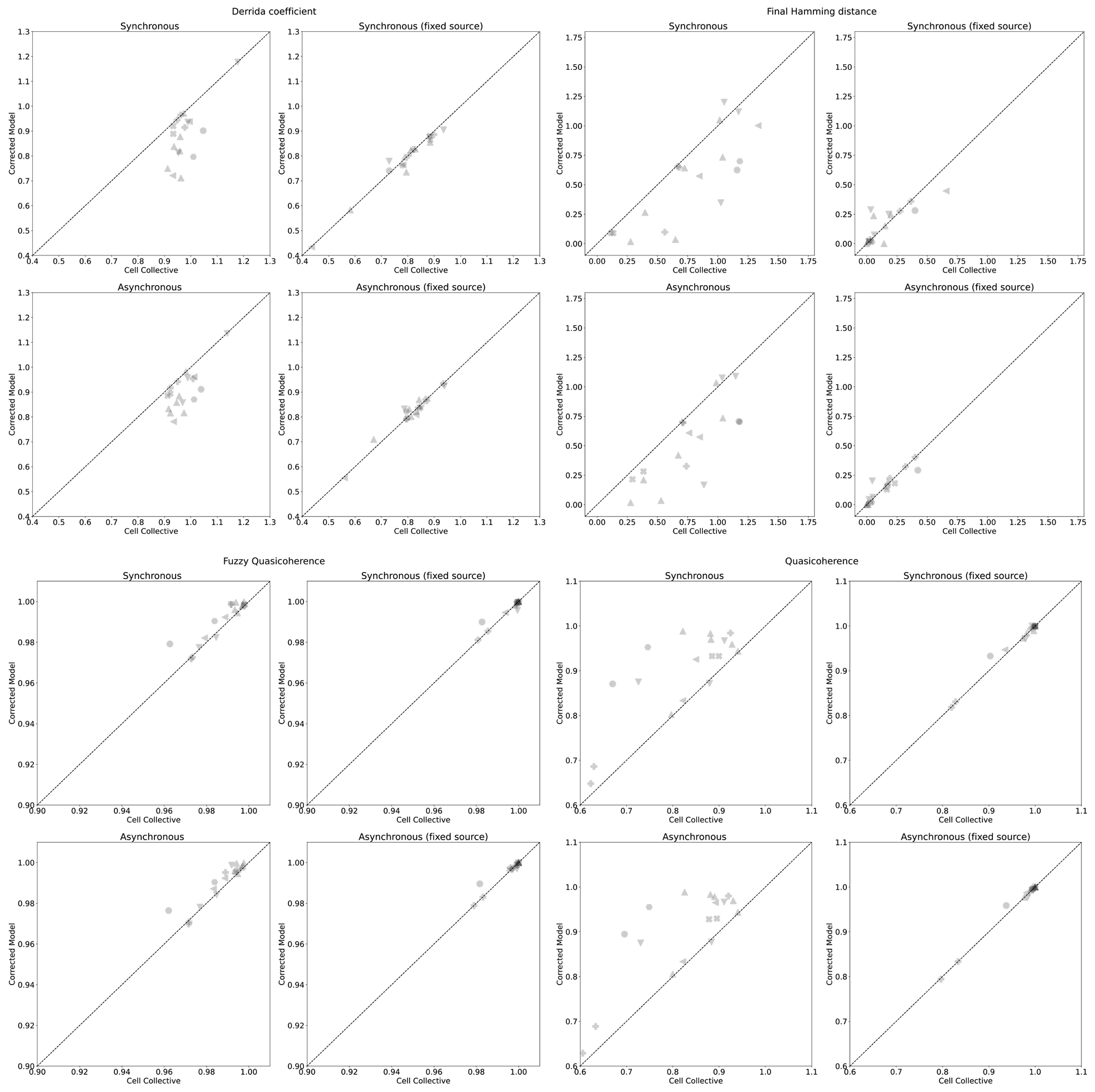
Comparison of key measures for the 18 models in the Cell Collective that were altered to attain a better agreement with the originally published models.

**FIG. 14.**
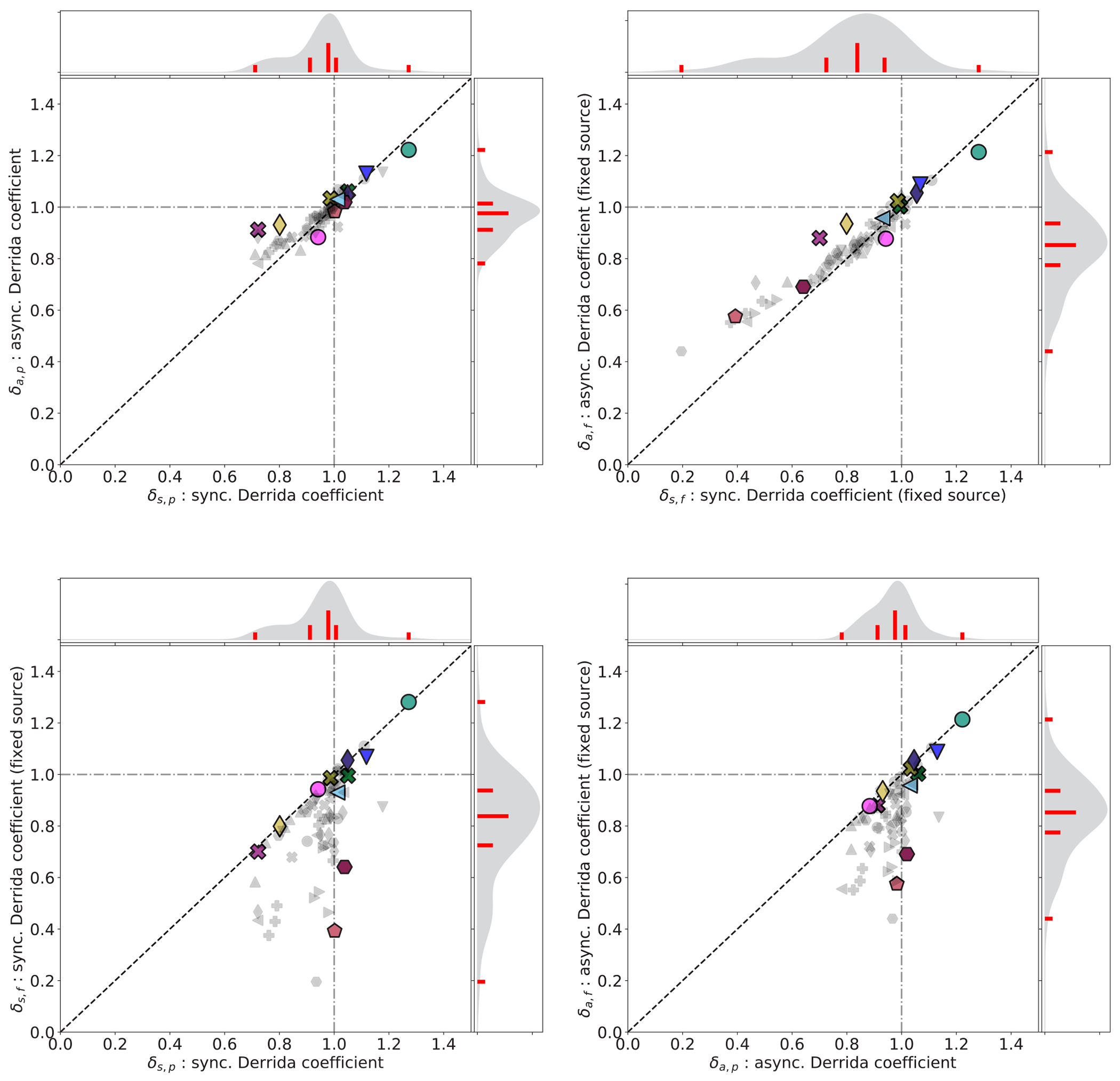
Systematic evaluation of the dependence of the Derrida coefficient δ on the update scheme and on source node perturbations. The ensemble of Cell Collective models shows a general agreement between the Derrida coefficients obtained for synchronous and asynchronous update (top panels). When source nodes not candidates for perturbation, the Derrida coefficient dramatically decreases (bottom panels). For example, note that three cancer drug models (plus signs) lie far from the diagonal in the lower two panels, indicating that these models are highly affected by perturbations to source nodes. This is to be expected, as the source nodes in these models represent known cancer drugs that were selected because they have a tremendous impact on the behavior of cancer cells.

In this section, we use two measures introduced in Sec. IID, namely the quasicoherence q and fragility φ, to illustrate that it is difficult to alter the long-term dynamics of trajectories using small, internal perturbations. We demonstrate, in Figs. 5 and 6, that careful comparison of the overall behaviors of perturbed and unperturbed trajectories reveals a higher degree of orderlike robustness than is observable using traditional measures alone. The bulk of this section is devoted to uncovering the mechanisms that underlie this previously hidden order in specific models. We identify three key factors that give rise to disagreement between our new measures and traditional measures: (i) the extreme potency of perturbations to source nodes, (ii) the presence of oscillatory attractors that can result in phase-shifted trajectories with the same long-term behavior, and (iii) higher sensitivity to update scheme in traditional measures.

The quasicoherence q describes the likelihood that a system undergoes a long-term phenotypic change in response to a small, transient perturbation. Higher q indicates a greater degree of phenotypic robustness (see Sec. IID). Note that the values of source nodes also contribute to the phenotype in this context, and so the effect of allowing source node perturbation is particularly pronounced for q. We find that overall, the distribution of q in the Cell Collective (Fig. 5) is highly concentrated near 1 for the fixed-source case (see also Fig. 17 in Appendix F). This indicates that it is relatively difficult to alter the phenotype of a functional module within a cell by perturbing a single internal component. Indeed, no model has greater than a 60% chance to change the quasiattractor due to perturbation to a random node; when source nodes are excluded from the set of perturbable nodes, this bound drops to just over 20%. An example of low quasicoherence is the Cortical Area Development Network (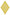) model [42], which has two attractors; the symbol lies on the diagonal because this model has no source nodes.

The distribution of q in the Cell Collective is fairly robust to update scheme, though there are exceptions. For example, note that Cell Cycle Transcription by Coupled CDK and Network Oscillators (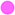) model has relatively low quasicoherence in the synchronous update, but a maximal quasicoherence in the asynchronous update (see Fig. 17 in Appendix F). The difference arises because the asynchronous update gives rise to only a single attractor (a steady state) while the synchronous update gives rise to an additional oscillatory attractor. In this case, the timing perturbations have interfered with the node perturbations in the system by destroying an attractor that is required for long-term separation of trajectories. The fragilities φ of the Cell Collective models also exhibit a distribution that is generally robust to the update scheme, and a shift to lower values when source nodes are not candidates for perturbation (see Fig. 18 in Appendix F).

**FIG. 15.**
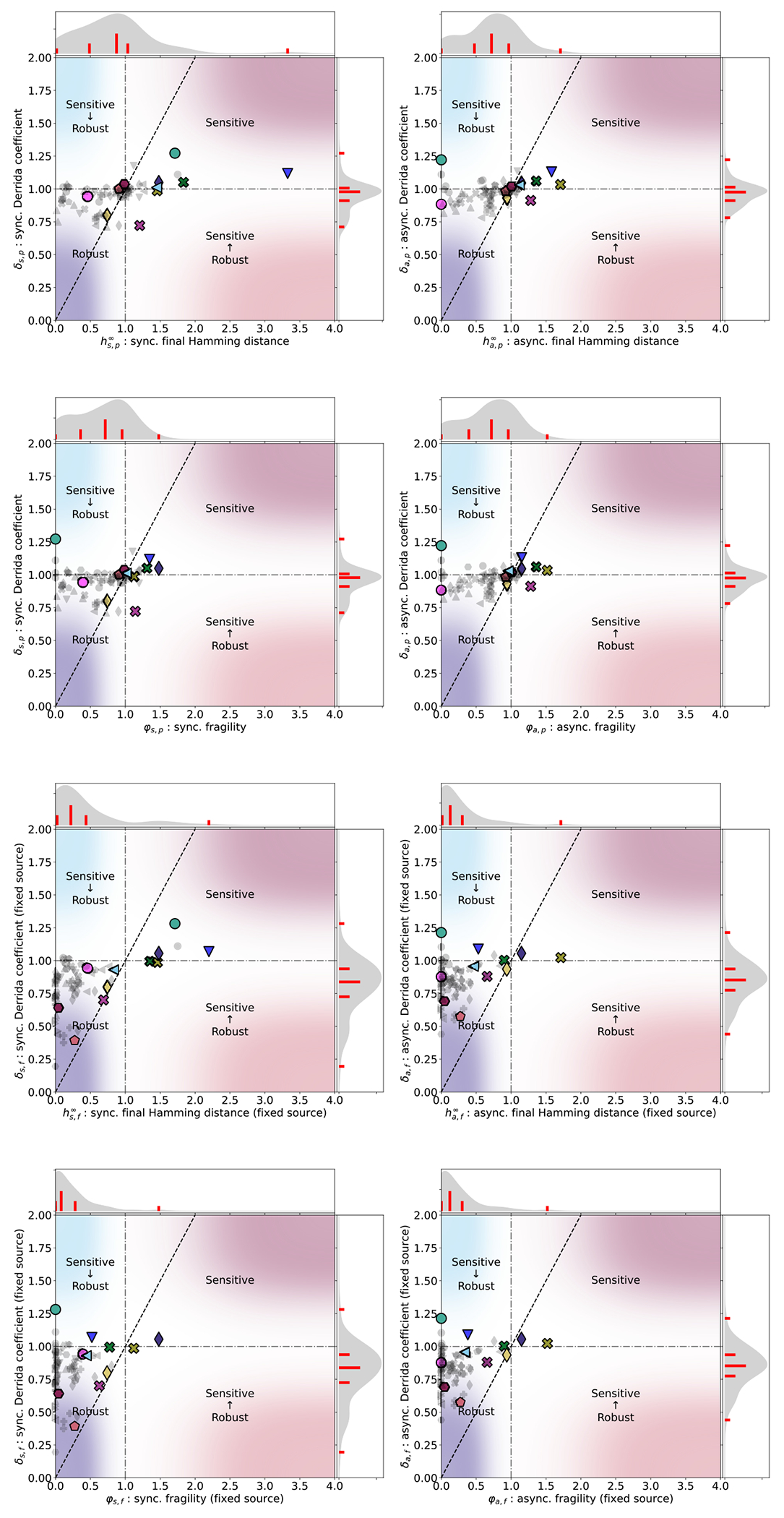
Relationships of the Derrida coefficient δ with the final Hamming distance $h^{\infty }$ and the fragility φ.

Separate from quantifying whether or not a perturbation induces a change in phase-shift-corrected long-term behavior (via q), we also quantify the magnitude of such changes using φ. Figure 6 summarizes the relationship between δ and φ under synchronous update with fixed source nodes. Note that only two models exhibit long-term perturbation growth (a hallmark of chaotic dynamics) once source nodes and phase shifts are accounted for, and the vast majority of the models are firmly in the robust regime of the φ distribution (associated with ordered dynamics). In contrast, the traditional analyses (e.g., [18,19,45]) place the majority of the models close to the critical boundary between the ordered and chaotic regimes, and also place several models in the chaotic regime (left panel of Fig. 4). We found no correspondence of δ with φ regardless of the manner of update or the perturbability of source nodes. Furthermore, unlike in the case of $h^{\infty }$, the φ distribution shows little dependence on the choice of update scheme. (See Fig. 15 in Appendix F for a comprehensive figure combining Figs. 4 and 6 with five other similar plots). This suggests that the ability of δ to predict long-term perturbation response is sensitive to phase-shifts and can overestimate the disruption a perturbation is likely to cause to a system’s phenotype.

As we illustrate with several examples below, it is often possible to reveal a robust order in apparently chaotic perturbation responses of specific functional modules by carefully analyzing the patterns of oscillation that perturbed trajectories undergo.

As highlighted previously in Fig. 4, the Signal Transduction in Fibroblasts (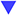) model [46] has a very high value of $h^{\infty }$ in the synchronous update (>3 when the source nodes can be perturbed and 2.3 when they cannot), and δ only slightly above 1. Asynchronous update decreases $h^{\infty }$, but $h_{a,p}^{\infty }$ and $h_{a,f}^{\infty }$ still indicate perturbation growth (see Fig. 15 in Appendix F). Due to the abundance of oscillating attractors in this model, large responses to perturbations may be expected. Despite this, φ is less than 1 in both update schemes in this model when source nodes are fixed, meaning that at the phenotype level, perturbations to individual nodes eventually decay on average. In other words, the majority of the perturbation response observed through the lens of $h^{\infty }$ is due to the effect of shifting the phase of a trajectory without altering its phenotype. The Aurora Kinase A in Neuroblastoma (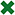) model is a smaller model that exhibits similar behavior.

**FIG. 16.**
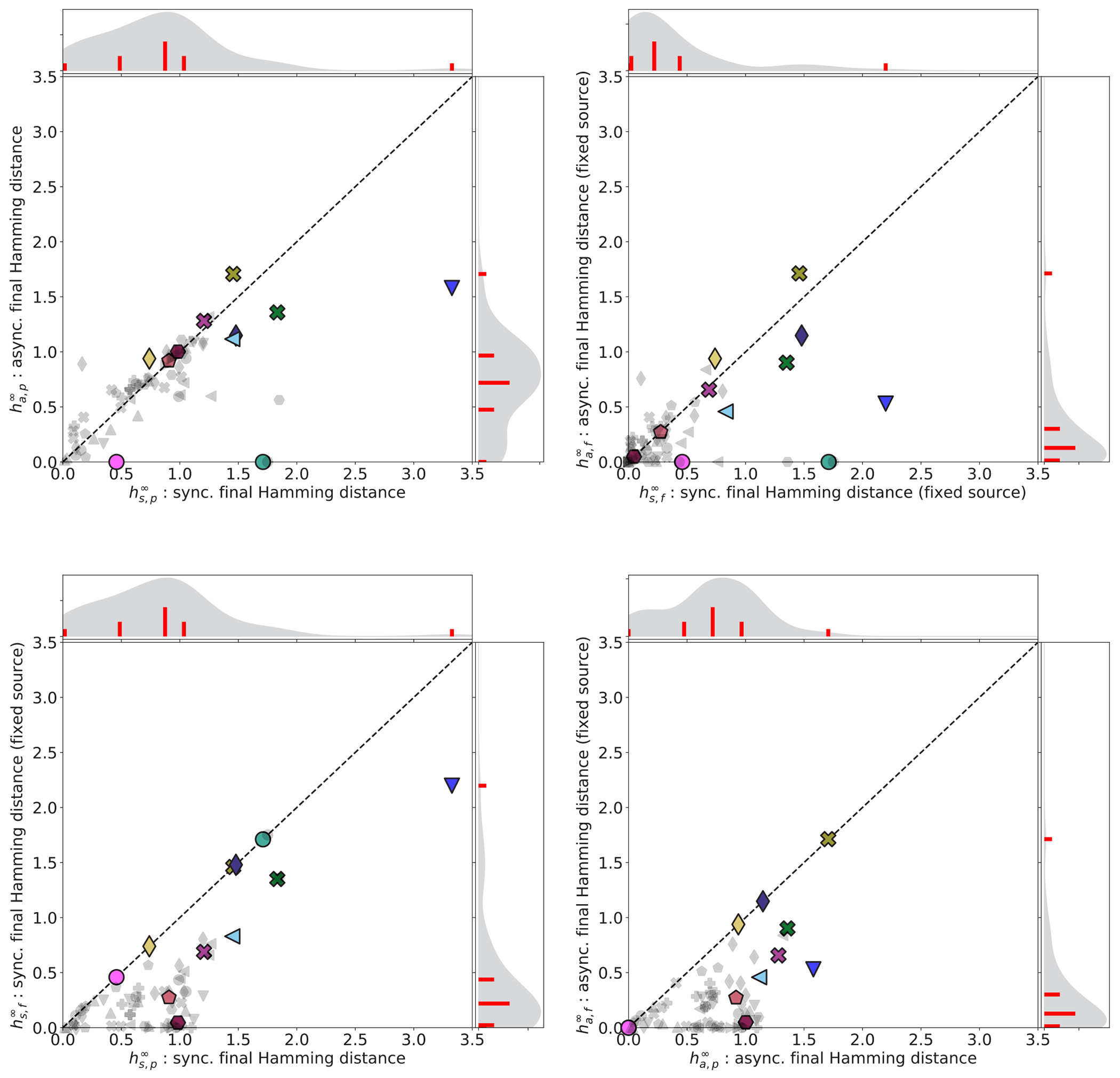
Comparison of different ways to compute $h^{\infty }$. The ensemble of Cell Collective models shows an overall agreement between the final Hamming distances obtained for synchronous and asynchronous update (top panels). Exceptions include models that exhibit significant phase shifts under synchronous update. When source nodes are not candidates for perturbation, the final Hamming distance dramatically decreases (bottom panels).

The *Arabidopsis thaliana* Cell Cycle (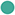) model [44] is also in the regime traditionally associated with chaos when synchronous update is used to compute δ and $h^{\infty }$ (Fig. 4), but a closer look reveals a robust phenotype. The original article reported an 11-state cyclic attractor under synchronous update, which recapitulates the phases of the cell cycle, and in which all 14 nodes oscillate. This model’s response to an initial perturbation to a single node is the highest observed (δ>1.2 in both update schemes). In the synchronous update, this initial separation persists, and even grows somewhat in the long term (reaching an average of over 1.7). Because there is only one attractor in this system, and because synchronous attractors are always simple cycles, this separation is due to a phase shift; indeed, the fact that the synchronous fragility of this model is zero reinforces this (Fig. 6). In the asynchronous update, both the fragility and the final Hamming distance are zero, indicating that this model exhibits a long-term robustness under the asynchronous update that is not detected by δ. The difference in long-term separation in the two updates reflects the fact that phase shifts are always permanent in the deterministic synchronous update, but can be temporary in the asynchronous update if there is an order of update that causes two trajectories in the same complex attractor to intersect. Indeed, there is a general tendency for a smaller final Hamming distance under asynchronous update than under synchronous update (see Fig. 16 in Appendix F). Furthermore, Fig. 19 in Appendix F suggests that phase-shifting behavior of the *Arabidopsis thaliana* Cell Cycle (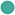) model is a common phenomenon; the final Hamming distance is always larger than or equal to the fragility in both update schemes, with an especially prominent difference in synchronous update.

There are two models that stay in the chaotic regime according to both $h^{\infty }$ and φ, the Human Gonadal Sex Determination (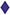) model [48], and the Colitis-associated Colon Cancer (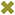) model [41]. These two are the only models with φ>1 when source nodes are not candidates for perturbation. The fragility of The Human Gonadal Sex Determination (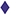) model is discussed in detail using a reduced version of the model in Appendix D.

In summary, our analysis of the Cell Collective models using our newly introduced measures of quasicoherence and fragility reveals that most of them are phenotypically ordered for both update schemes considered. With these measures, we uncover nontrivial perturbation recovery on long timescales even in putatively chaotic perturbation responses captured by the final Hamming distance, and we identify key mechanisms behind phenotypic fragility and robustness.

## IV. DISCUSSION

One of the conjectured hallmarks of complex biological systems is that they sit somewhere between rigid order and hypersensitive disorder. For example, a yeast cell must be able to adjust its metabolic phenotype in response to external cues such as oxygen availability, and to internal cues that operate downstream of cellular mechanisms involved in processing environmental signals. At the same time, the yeast cell must not chaotically switch between metabolic pathways in response to small fluctuations in external conditions or in response to noise in its internal regulatory processes. From an evolutionary perspective, some degree of phenotypic mutability confers adaptability to a population; too much leads to a lack of evolvability or even population collapse [49]. It has been argued that in living systems, there is often a sharp boundary between these regimes, and the cusp of this boundary is the ideal place to balance these competing needs [1,4–6,10,18]. Indeed, in simple random models that resemble biomolecular regulatory systems, this appears to be the case [5,6,17,28]. The argument is further bolstered by the fact that real-world models of specific within-cell functional modules share some properties exhibited by these simple random models in the critical regime [15,19,50,51].

But these real-world models are not random; for instance, they exhibit a higher degree of canalization and functional redundancy [19,52,53], as well as a higher occurrence of source nodes (as demonstrated here). Of course, it is well-known that these models are nonrandom, and researchers are typically careful to acknowledge the caveats this entails. For example, Kauffman considers the question of random network assembly in some depth from a biological perspective [4]; Moreira and Amaral give a rigorous treatment of the implications of nonergodicity and canalization in Boolean ensembles [53]; Zañudo and colleagues give a careful treatment of the underlying assumptions of randomness and their implications [28]; and we ourselves have discussed the potential pitfalls of applying techniques designed for random networks to nonrandom networks in previous work [15,19]. The Derrida coefficient [5,6], or its close cousin, the network sensitivity [10], are superb tools in the setting in which they were developed: synchronously updated random models. In that setting, they offer a computationally simple way to determine the short-term and long-term response of the system to perturbations. Even in nonrandom models, these tools remain valid for exploring the short-term perturbation response, and they can be extended to focus on steady-state robustness (e.g., by extending the influence measure of [54]), but more sophisticated measures are required for studying their long-term dynamics in response to perturbations.

The traditional approach to directly quantifying the long-term response to perturbations is to measure what we have called the final Hamming distance. This measure provides valuable information about the asymptotic separation of perturbed and unperturbed trajectories, but fails to account for time-shifts. By considering whether perturbed and unperturbed trajectories differ in ways that are in principle observable under typical experimental settings, the new measures we introduce provide a phenotypically grounded way to quantify the ultimate impact of a perturbation. Our analysis shows that the responses to internal perturbations that have been previously associated with criticality are usually either more transitory than initial perturbation growth may suggest or become phenotypically irrelevant in the long term. In fact, in the studied experimentally supported, nonrandom models we uncover much greater robustness to perturbation, especially in their long-term effects, than the criticality hypothesis implies.

Though such orderly behavior of functional modules (cell processes) has been overlooked, indeed hidden by the typical measures of criticality used, it is not altogether surprising. For example, it is fundamental to Kauffman’s thesis that orderly behavior can arise naturally from RBNs [3,4] and may play a key role in the evolution of epigenesis. More recent work [45] has analyzed microarray time-series data to suggest that eukaryotic cells do not lie in the chaotic dynamical regime. Particularly at the scale of individual functional modules, we would expect a high degree of reliability in task execution under most perturbations. For example, to effectively balance photosynthesis efficiency with water conservation, the regulatory mechanism of stomatal guard cells in plant leaves must reliably respond to stress hormones produced by other modules in the plant’s regulatory network. Indeed, we observe that in the Guard Cell Abscisic Acid Signaling model [55] and the Stomatal Opening Model [56], the fixed-source fragility is quite low (see Appendix G). In contrast, the traditionally used Derrida coefficient suggests functional modules near or in the chaotic regime. We interpret this to suggest that small errors in signal transduction may lead to large initial deviations in these systems, but that eventually these errors are corrected in most cases. In the context of cell differentiation, Waddington [57] argues for a kind of long-term developmental robustness referred to as canalization; once committed to a cell fate, it is expected that a stem cell is not easily diverted from its specialization. We observe this in various development and differentiation models, such as the Lymphoid and myeloid cell specification and transdifferentiation model [58]. In this model, the short-term perturbation response suggests criticality $\left(\delta_{s,p}=1.02\right)$, but a long-term view reveals that initially divergent perturbed trajectories are canalized toward the fate of their unperturbed counterparts in most cases ($q=0.9_{s,p},\varphi_{s,p}=0.16$).

The new measures we introduced to characterize this robustness or phenotypic order allow us to distinguish process delay from phenotype differentiation $\left(h^{\infty }\right.$ versus φ), and to separate smoothly varying distance in -omics space from “all-or-nothing” phenotype differences (φ versus q). These measures are computationally expensive to estimate, and until now, their estimation on ensembles of large models (more than a few dozen nodes) has been prohibitive. Here, we have addressed this challenge by developing cubewalkers, a highly parallel GPU-based simulation toolkit. Our analysis showcases its capacity for comprehensive calculation of long-term perturbation dynamics in real-world Boolean networks with hundreds of nodes or more. Future work will consider these measures in the context of random Boolean networks. Together with traditional measures, our new approaches offer a more holistic way to study the dynamical response of living systems to noise and perturbation.

Though our analysis suggests that the criticality of experimentally supported Boolean models of biomolecular functional modules has been overstated, we emphasize that this work is not the nail in the coffin of the “edge of chaos” hypothesis. Rather, it suggests that living systems do not exhibit critical behavior *at the scale of functional modules*. This leaves ample room for critical behavior to emerge at larger scales via the coupling of various functional modules. Indeed, previous work by Balleza and colleagues [18] suggests cell-scale critical perturbation response in two full-genome regulatory networks with experimentally constrained topology and random regulatory functions, though the authors do not consider phase shifts in their analysis. We conjecture that individual subsystems of a cell are highly ordered, but they connect in networks that may give rise to more adaptive behavior. The large differences in perturbation response we have observed depending on the treatment of source nodes (which are exceedingly rare in traditional RBN models) support this conjecture because it allows for larger perturbation responses in networks of highly ordered functional modules coupled at their source nodes. In critical RBNs, one may view the nodes themselves as ordered subsystems. In real biological systems of many variables, a multiscale, modular structure is expected [59]. Thus, it is possible that order persists up to larger scales in biology than it does in random models. More thorough examination of criticality and perturbation response across regulatory scales is needed to test our conjecture, which motivates the future development of sufficiently data-constrained multiscale models.

Despite our finding that the Derrida coefficient is not a good predictor of phenotypic robustness, we do not suggest that it is without merit in models of specific functional modules. Instead, we merely caution that it must be carefully interpreted as an indicator of *immediate* response to perturbation only and should be studied in conjunction with long-term response measures, such as those we have developed here. We do suggest, however, that careful consideration be made to the biological interpretation of source node perturbation in the context of the particular network being considered. Generally, we advise that perturbation of these nodes be handled separately from perturbations to other nodes in the network.

We have also studied timing perturbations in these systems by considering the effect of update scheme on various dynamical properties. Many update schemes exist for Boolean networks, such as the most permissive Boolean network framework of [60], random order update [30], or various update schemes that make use of a continuous time parameter such as is used in MaBoSS [61]. We focused on the synchronous update and the asynchronous update, which are the most frequently used and are the two opposite extremes of the spectrum from deterministic timing coherence to completely stochastic event timing. Models with long-term perturbation growth under synchronous update also appear to be more sensitive to timing perturbations (comparing the highlighted models in Figs. 3 and 4). This is possibly related to the fact that a single-node perturbation can be interpreted as an asynchronous modification to the perturbed node. Previous work [26] has shown that certain patterns of logical circuitry, called conditionally stable motifs, can help explain robustness to timing perturbation in some cases and may also confer perturbation robustness. Such robustness is not guaranteed, however. It is well established that the update scheme can have a dramatic impact on the attractor dynamics of Boolean networks (see, e.g., [25]). In the models considered here, the average behavior of individual system components is typically quite robust to update scheme, but in a few models there is a dramatic difference in the biological interpretation of the individual trajectories that are possible in one update scheme or the other. In the examples we have examined here where this is the case, there are attractors that exist in the synchronous update but which are absent in the asynchronous update. In all such cases, the attractors were motif-avoidant, i.e., they did not fall into any minimal trap space [25] (sometimes these are called unfaithful attractors [62]). In these examples, delay nodes played a prominent role in the behavior of the model under synchronous update.

**FIG. 17.**
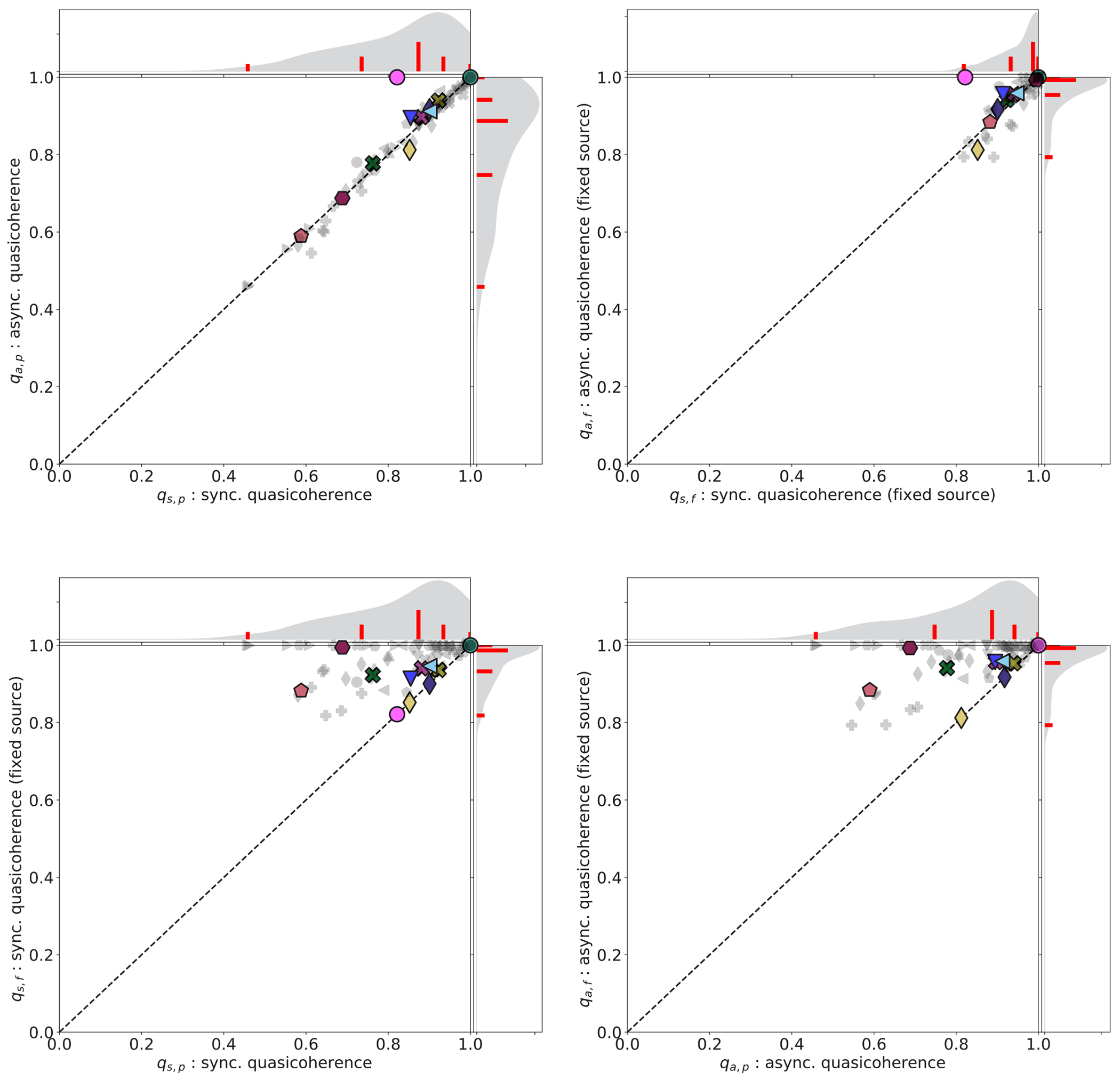
Comparison of different ways to compute q. There is a general agreement between the quasicoherences obtained for synchronous and asynchronous update (top panels). Making the source nodes not candidates for perturbation dramatically decreases the fragility (bottom panels).

**FIG. 18.**
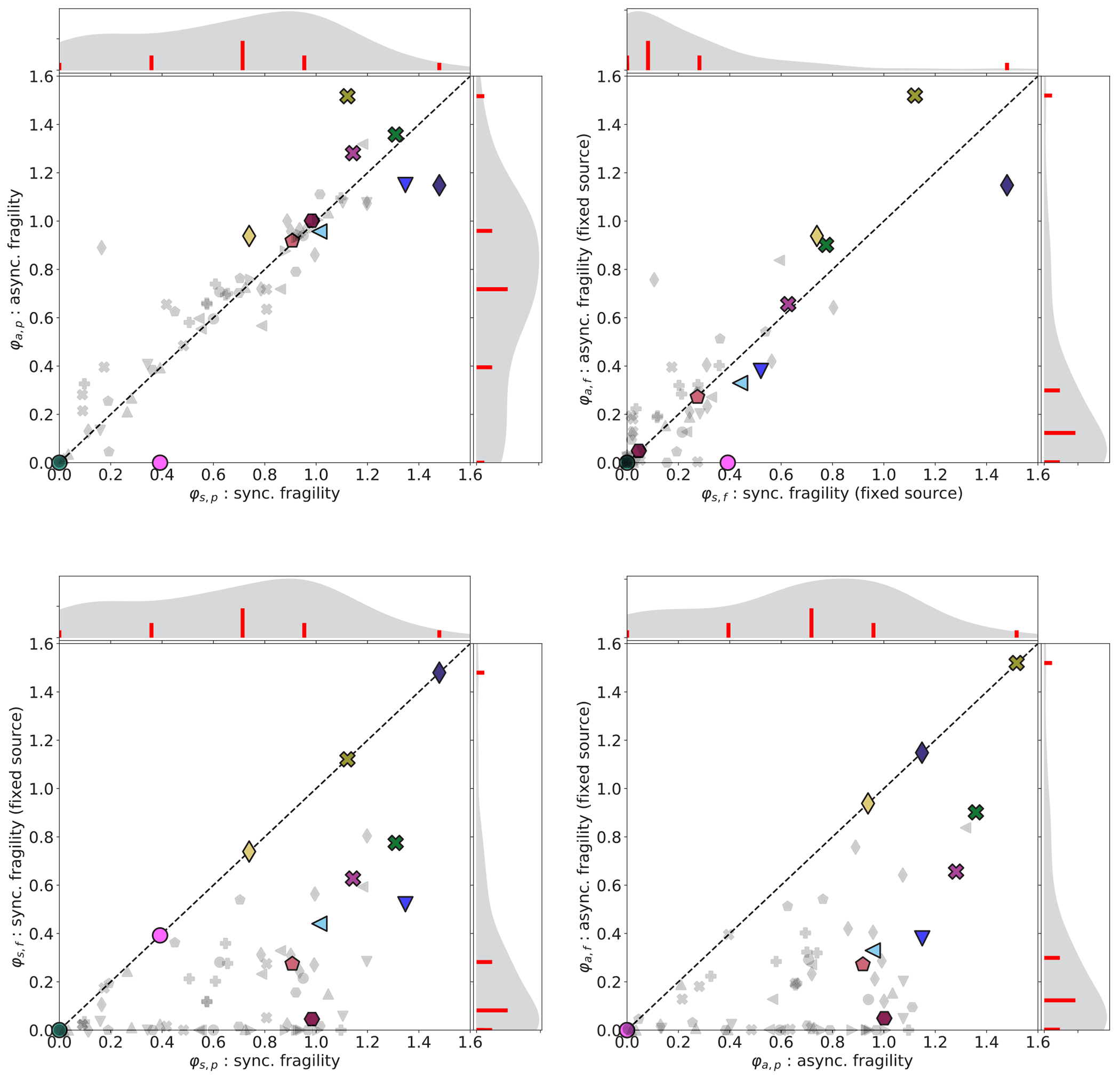
Comparison of different ways to compute φ. There is a general agreement between the fragilities obtained for synchronous and asynchronous update (top panels). Making the source nodes not candidates for perturbation dramatically decreases the fragility (bottom panels).

**FIG. 19.**
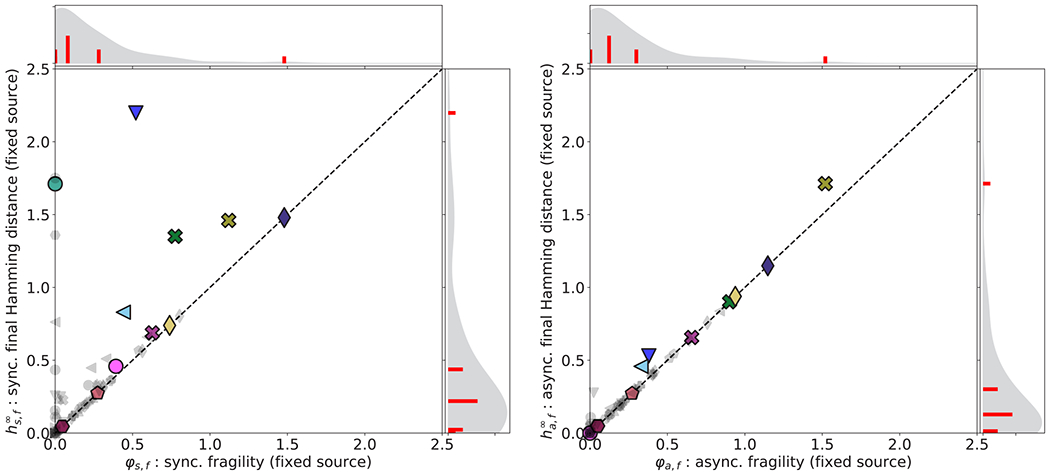
Comparison of the final Hamming distance $h^{\infty }$ and the fragility φ. The final Hamming distance is always larger than or equal to the fragility in both update schemes. The difference is much more prominent in the synchronous update in which any phase shift is permanent, compared to the asynchronous update in which the stochasticity can disperse it.

We generally found that models appear more ordered in the asynchronous update, for example via the destruction of synchronous attractors. Most dramatically, the median value of $h^{\infty }$ for fixed source nodes is approximately 43% higher in the synchronous update than in the asynchronous case. We conjecture that noise in the update timing can suppress the phase-dependent effects of node perturbation. Indeed, while two phase-shifted oscillating trajectories can never realign in the synchronous update, eventual realignment is likely under the asynchronous update. Thus, the long-term response to node perturbations becomes biased toward extinction in the asynchronous update as measured by $h^{\infty }$ (see Fig. 16 in Appendix F). In contrast, because q and φ inherently account for phase-shifts in perturbed trajectories, they are much less sensitive to update scheme (see Figs. 17 and 18 in Appendix F).

Though we have briefly examined the time dependence of the Hamming separation $h^{t}$, much about perturbation response on intermediate timescales remains unexplored. In some models, transient behaviors play a crucial role in the biological interpretation of trajectories. For example, in [63], a cell cycle model is presented in which the ultimate fate of any asynchronously updated cell is death. Despite this, trajectories exhibit behavior that is similar to experimentally observed processes. Analyzing such a model using the framework we have presented here would require modifying truncating the time averaging to capture phenotypically relevant periods prior to apoptosis.

We have illustrated the overall patterns observed in the experimentally supported model ensemble by carefully examining the dynamics of specific examples and considering dynamical behavior in the context of their intended biological modeling goals. This has highlighted that the rich diversity of biological function is not easily distilled to a few statistical properties. Some functional modules have dynamics that almost trivially follow from the configuration of their inputs, while others modules are highly multistable with long-term dynamics that depend strongly on initial conditions and internal timings. In the search for unifying principles in biology, it is important to acknowledge that biology is messy and that functional context matters—especially in the study of specific subsystem models. In other words, living systems are complex, open systems. While there are important general conclusions we can draw, the differences between biomolecular systems can be just as interesting as their common properties. In that spirit, we show that functional modules in biomolecular systems typically exhibit robust phenotypes, while highlighting the diverse mechanisms through which this hidden order can arise. The observed order, as a phenomenon of experimentally supported models, has been hitherto obscured by the lack of dynamical measures that can quantify it and the computational challenges of measuring the dynamics with sufficient detail, an obstacle we overcame in the present work.

We hope that as computational biology continues its second half-century, unprecedented computational power allows deeper exploration of the interplay between order and chaos in living systems, and helps uncover the unique biological circumstances that enable it.

## Figures and Tables

**FIG. 7.**
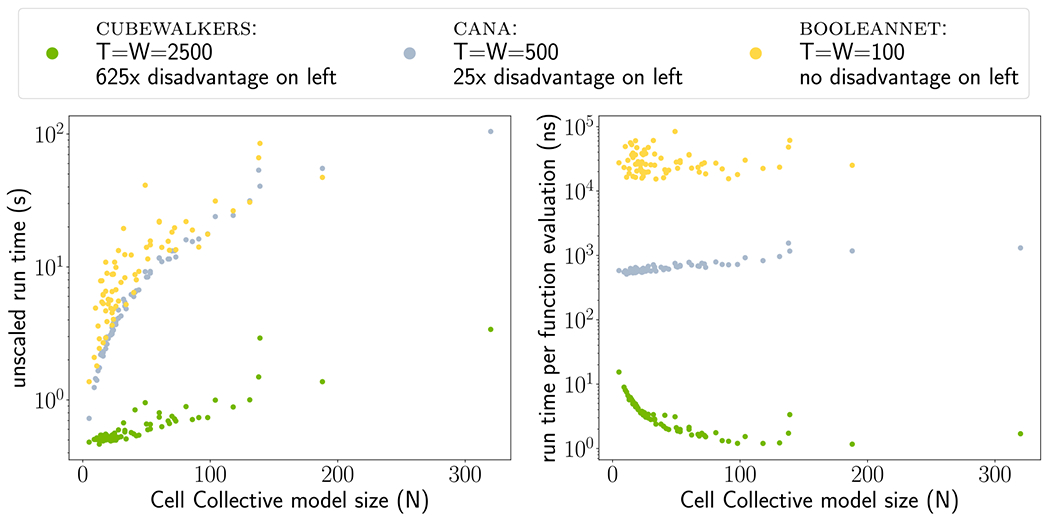
Performance comparison of cubewalkers, cana, and booleannet on consumer hardware. 72 Cell Collective models were run using each tool using synchronous update. Timings were generated on a PC with an AMD Ryzen 53600X CPU at 3.8GHz and a 2560 CUDA-core 1605 MHz NVIDIA 2070S GPU. Default methods were run without additional parallelization. For the cubewalkers tests, 2500 time steps and 2500 walkers (initial conditions) were used; for cana, 500 time steps and 500 walkers were used; and for booleannet, 100 time steps and 100 initial conditions were used. Thus, for each network, cana computed 5× as many time steps for 5× as many initial conditions as booleannet for an overall disadvantage of 25×. Similarly, cubewalkers computed 5× as many time steps for 5× as many initial conditions as cana, for a 25× disadvantage relative to cana and a 625× disadvantage relative to booleannet. The raw time to complete these tasks is plotted in the left panel, where we observe that cubewalkers consistently finishes its tasks an order of magnitude faster than the other methods, despite the fact that it has been given significantly more computational work. In the right panel, the average computation time per network node per time step per initial condition in these trials is plotted; this corresponds to the average (amortized) time to evaluate and apply an update function to a node. Here, we see that these amortized evaluations occur on the order of nanoseconds for cubewalkers, while they occur on the order of microseconds for cana and hundreds of microseconds for booleannet.

**FIG. 8.**
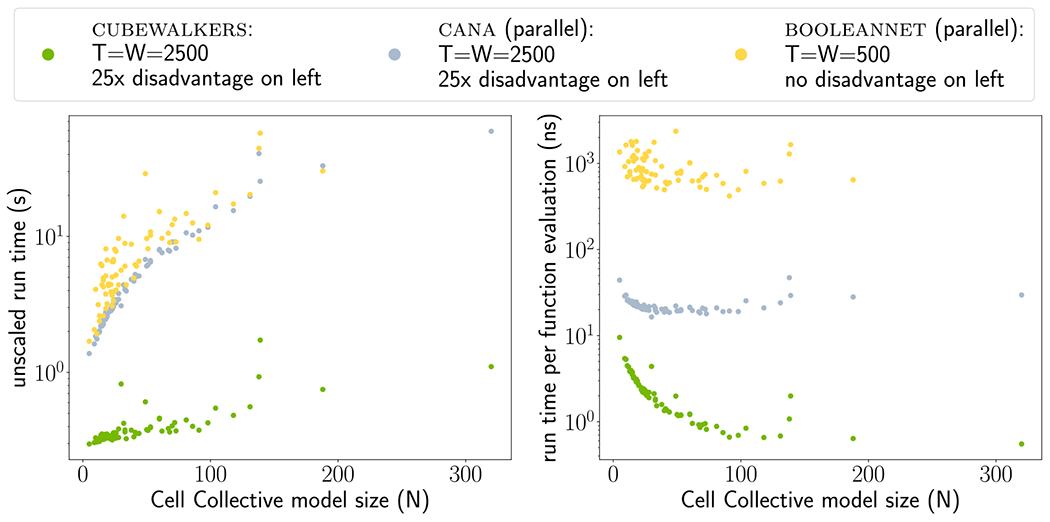
Performance comparison of cubewalkers, cana, and booleannet on a high-performance computer. Cell Collective models were run using each tool using synchronous update. Timings were generated using a workstation with two AMD EPYC 7542 CPUs (32 cores and 64 threads each) at 2.9GHz and two 10 752 CUDA-core NVIDIA A6000 GPUs with 48GB of GDDR6 memory (only one GPU was used for the benchmarks). For the cubewalkers and cana tests, 2500 time steps and 2500 walkers (initial conditions) were used; for booleannet, 100 time steps and 100 initial conditions were used. For cana and booleannet, initial conditions were simulated in 128 parallel threads. On specialized hardware taking full advantage of parallelism, we see that the performance gap between cubewalkers and the other methods is narrowed compared to the performance gap on consumer hardware. Nevertheless, the gap remains considerable.

**FIG. 9.**
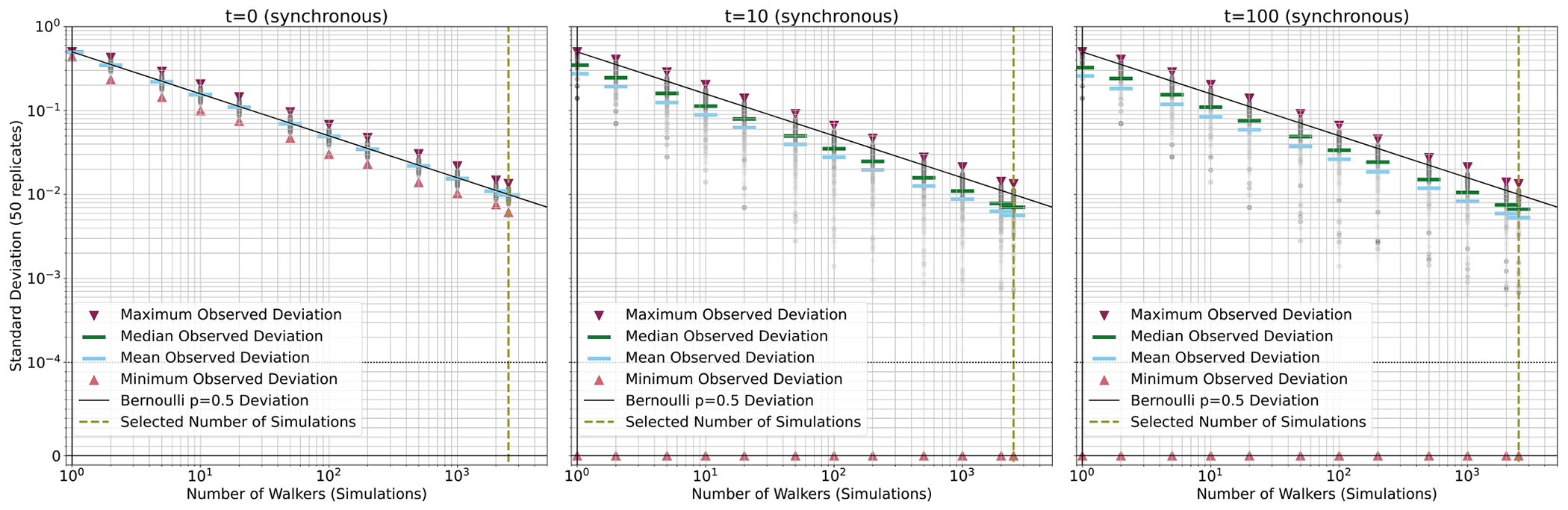
Standard deviation in the node average values as the number of walkers increases for the Cell Collective models. The three panels correspond to three different stages of the model simulation. The observed standard deviations agree well with the expectation based on Bernoulli random variables (continuous line). We chose the number of walkers such that the standard deviation is less than 0.01 (dashed vertical line).

**FIG. 10.**
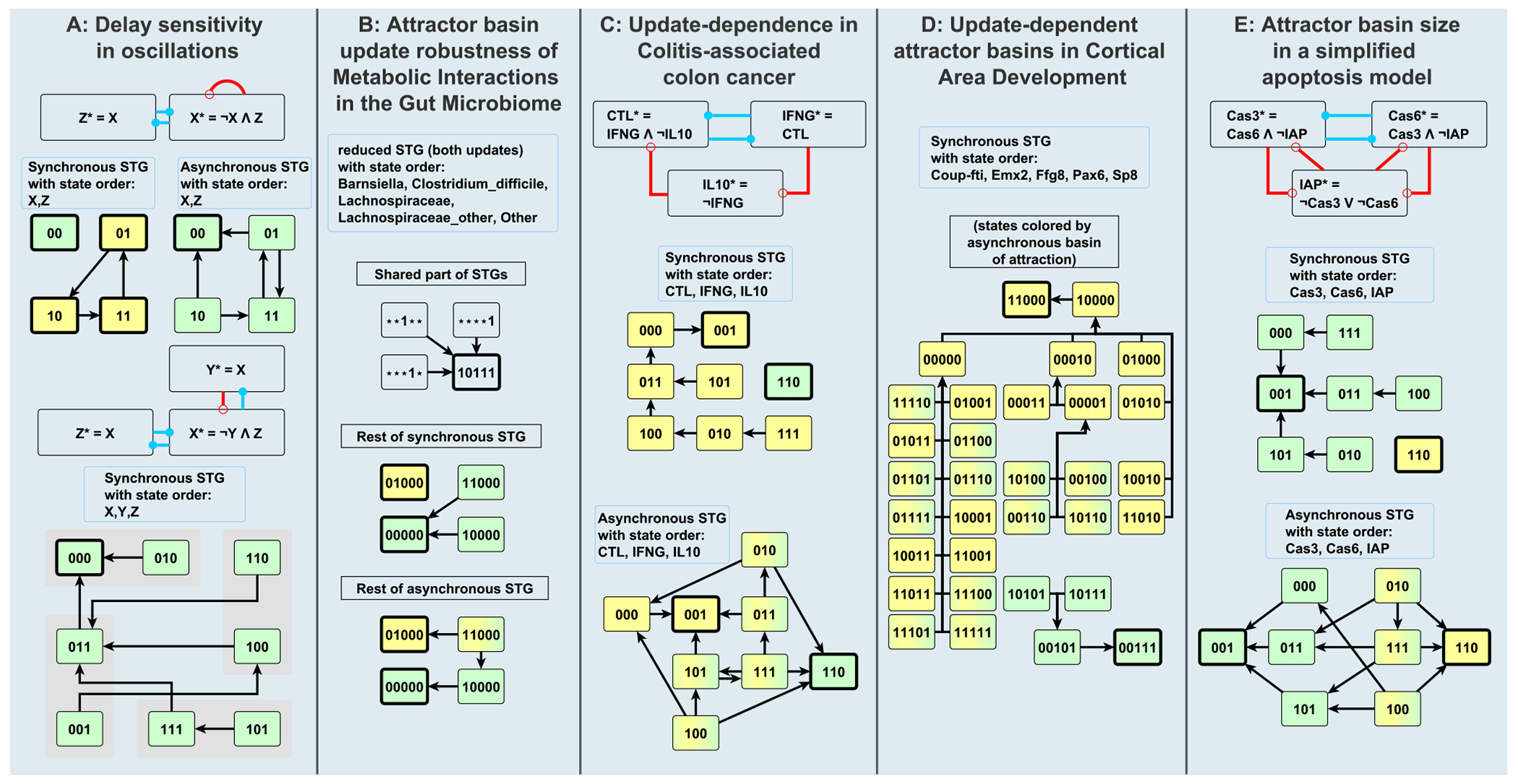
Example networks and their state transition graphs (STGs) that illustrate update dependence. Panel A illustrates delay-dependency in the example of Eqs. (D1) and (D2), which are inspired by the Cell Cycle Transcription by Coupled CDK and Network Oscillators model [36]. Panel B demonstrates how much of the STG in the Metabolic Interactions in the Gut Microbiome model [40] is robust to changes in update scheme. Panels C–E illustrate how the asynchronous update can mix the synchronous attractor basins in a core regulatory circuit in the Colitis-associated colon cancer model [41] (panel C), the full synchronous STG of the Cortical Area Development model [42] (panel D), and a reduced version of the Apoptosis Network model [43] (panel E). In each interaction network, each node symbol contains the update function of the node. Blue edges ending in filled circles indicate positive regulation, and red edges ending in open circles denote negative regulation. In the STGs, attractor states are indicated by thick borders. The basin of attraction of each attractor is highlighted by the same color as the attractor. In asynchronous update, states can reach more than one attractor; such states are shaded using a gradient. In the lower part of panel A, states that differ only in the value of the delay node *Y* are grouped together in shaded boxes.

**FIG. 11.**
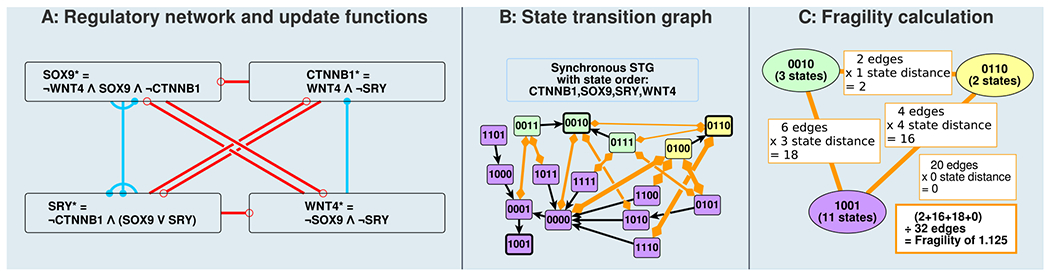
Fragility of a four-node reduced version of the Human Gonadal Sex Determination model of [48]. Panel A depicts the interaction network. Each node symbol contains the update function of the node. Blue edges ending in filled circles indicate positive regulation, and red edges ending in open circles denote negative regulation. Panel B shows the state transition graph under the synchronous update. Attractor states are indicated by thick borders. The basin of attraction of each attractor is highlighted by the same color as the attractor. State transitions are shown with black arrows, and orange double-sided arrows indicate state pairs that are related by single-node perturbations. These are the transitions that can arise from single-node perturbations and that lead to different long-term behavior than is observed without perturbation. The thickness of each orange edge indicates the Hamming distance between the corresponding attractors. Panel C shows how to calculate the fragility of this reduced model exactly using the information in panel B.

## APPENDIX A: BENCHMARKS

In this Appendix, we present benchmarks comparing the cubewalkers software to two competing software packages: cana and booleannet (see Figs. 7 and 8). Conducting unbiased quantitative benchmarks that compare the performance of cubewalkers to that of other Boolean simulation tools is complicated by the fact that cubewalkers is primarily GPU-based, while competing tools run entirely on the CPU.

**TABLE I.** Average (amortized) run time per simulation time step for each method. The fastest method (cubewalkers) for the two hardware configurations is bolded. Note that cubewalkers on consumer hardware outperforms parallel adaptations of other tools running on specialty high-performance computing hardware.

Software	Hardware	time (μs)
cubewalkers	consumer	**0.11**
cubewalkers	specialty	**0.067**
cana (serial)	consumer	40
cana (parallel)	specialty	1.1
booleannet (serial)	consumer	1300
booleannet (parallel)	specialty	37

Therefore, one must assess the relative quality of the CPU and GPU used in benchmarking comparisons. Furthermore, most CPU-based Boolean simulation tools do not execute operations in parallel; however, user-side parallelization is often possible. Despite these caveats, the performance advantage of cubewalkers is dramatic and convincing in practice. We compared against the Python library cana (which wraps a C implementation via Cython) [31] and the Python library booleannet (which is written in pure Python) [30]. For synchronous simulations of models in the Cell Collective on consumer hardware, we demonstrate a speedup of approximately 350 times on average compared to simulation using cana and a speedup of approximately 11 000 times on average compared to simulation using booleannet. We also compared the performance of cubewalkers to the performance of parallelized simulations using cana and booleannet on a high-performance computing workstation. In this case, cubewalkers outperforms cana by a factor of 16.6 and outperforms booleannet by a factor of just under 550. Furthermore, cubewalkers has approximately 10 times better performance on our consumer test hardware than is achieved using parallel simulations with cana on our specialty high-performance hardware. Average performance is described in Table I. Note that the simulation results presented herein required several days of computer time using cubewalkers, so the approximately 16-times slowdown we expect from the second-fastest software considered would result in months of excess computation.

## APPENDIX B: CONVERGENCE OF AVERAGE NODE VALUES

The number of walkers were selected to ensure a standard deviation of less than 0.01 for each dynamical measure computed. The minimum simulation count of W=2500 was used in the calculation of average node values. The convergence of these values as a function of W is shown in Fig. 9. For Derrida coefficient calculation, a value of approximately W=100 000 was used (W=⌊100 000/N⌋×N); for measuring long-term perturbation spread, a value of W=2500 was used for each node targeted for perturbation (for a total of 2500×N simulations each, resulting in W=800 000 in the largest model considered).

The question of how many time steps are required to have a reasonable expectation of average node value convergence is more complicated. There are two reasons for this: (i) convergence time is highly model-dependent and (ii) as the systems considered are generally not ergodic, the average node values may converge into oscillatory behavior Thus, there are two parameters that need to be considered: a “burn-in” time $T_{b}$, and an averaging time window size $T_{w}$, for a total simulation time of $T=T_{b}+T_{w}$. We fixed $T_{b}=50N\;+\;1000$, so that at least 1000 updates are performed and each node is updated more than 50 times on average in the asynchronous update during the burn-in stage. We then varied $T_{w}$ and evaluated the convergence of the average node values by comparing the values calculated in four subwindows: $T_{wi}=\left[T_{b}+iT_{w}/5,T_{b}+(i+2)T_{w}/5\right]$ for i=0,1,2,3. For each network in the Cell Collective, we computed the absolute difference in average node values for each of the six pairs of these four subwindows, and we identified the largest absolute difference across all six comparisons for each node. Convergence quality is assessed by computing the largest of these values across all nodes. Based on this analysis, we chose to use a value of $T_{w}=5N\;+\;5000$ for most models. Three models took an unusually long number of time steps to converge due to the complexity of their attractors; for these we set the number of time steps manually: $\left(T_{b},T_{w}\right)=(5000,\;25 000)$ for “Arabidopsis thaliana Cell Cycle” (N=14), $\left(T_{b},T_{w}\right)=(5000,\;25 000)$ for “Guard Cell Abscisic Acid Signaling” (N=44), and $\left(T_{b},T_{w}\right)=(50 000, 100 000)$ for “Signal Transduction in Fibroblasts” (N=139). The largest absolute difference of average node values between any two time subwindows $T_{wi}$ and $T_{wj}$ across all nodes in all networks was approximately 0.0004 in the synchronous update and 0.0066 in the asynchronous update. Summing the largest difference for each node gives a maximum of 0.0039 and 0.0881 for synchronous and asynchronous update, respectively, across all networks in the Cell Collective. The actual computed quantities aggregate many nodes and average over a time window 2.5 times larger than any $T_{wi}$; thus, in practice, they have errors much lower than this very conservative upper bound. We are therefore confident that simulating each network for T=55N+6000 time steps and averaging node values over the last $T_{w}=5N\;+\;5000$ time steps is sufficient for computing the average behaviors of nodes in almost all models in the Cell Collective.

## APPENDIX C: THE RELATIONSHIP BETWEEN TWO DYNAMICAL MEASURES: FUZZY QUASICOHERENCE AND FRAGILITY

The quasicoherence measure treats trajectories that converge to the same quasiattractor as equivalent, even if they converge to different attractors within that quasiattractor. We introduce the fuzzy quasicoherence, a modification of the quasicoherence such that it becomes sensitive to the similarity of attractors but retains phase-insensitivity. This is achieved by replacing the Q function with a “fuzzy” version that considers the absolute difference between X(t) and $X^{\left(\neg i\right)}(t)$. This gives rise to the fuzzy quasicoherence, $\tilde{q}$:

(C1)
$$\tilde{q}={\langle \frac{1}{N}\sum_{i=0}^{N-1}\tilde{Q}{\left(\langle X(t)\rangle \right)}_{t\to \infty },{\langle X^{\left(\neg i\right)}(t)\rangle }_{t\to \infty }\rangle }_{X\in 𝒯},$$

(C2)
$$\tilde{Q}(X,Y)=1-\frac{1}{N}∥X-Y{∥}_{1}.$$

Note the similarity with both q and $h^{\infty }$. Compared with q, the formula for $\tilde{q}$ replaces the Q function with $\tilde{Q}$, which, like Q, is 1 if the inputs are equal and 0 if the inputs are maximally different in each entry, but which can interpolate between 0 and 1. The ability to interpolate between the extremes of Q allows $\tilde{q}$ to account for whether quasiattractors are similar or different, and it also allows $\tilde{q}$ to account for attractors within the same quasiattractor that have different average node level behaviors. Compared with $h^{\infty },\tilde{q}$ can be viewed as a rescaling with a slightly modified averaging scheme.

The fragility is related to the fuzzy quasicoherence by the relationship $\varphi =N(1-\tilde{q})$.

## APPENDIX D: DETAILED DISCUSSION OF SELECTED REPRESENTATIVE MODELS

### 1. Cell Cycle Transcription by Coupled CDK and Network Oscillators

The Cell Cycle Transcription by Coupled CDK and Network Oscillators () model [36] incorporates the known interactions among nine cell cycle transcription factors and is one of several variants studied by Orlando *et al.* In synchronous update, this model has a point attractor corresponding to the G0 checkpoint and an oscillatory attractor that reproduces the sequence of transcription during the phases of the cell cycle. We find that the oscillatory attractor disappears under asynchronous update. This result indicates that the model can only reproduce the biological sequence of events if the node states change in synchrony.

To better understand the mechanisms that lead to this timing perturbation sensitivity, we simplified the model by merging closely related nodes and verified that the simplified model reproduced the correct transcription sequence under synchronous update. A key feature of the resulting network is that it consists of a positive feedback loop that intersects a shorter negative feedback loop. In general, this property ensures that the system is not monostable under synchronous update [67], i.e., an attractor other than the G0 fixed point exists. This extra attractor relies on synchrony and is therefore not robust to timing perturbation. The simplest example with these features is given in Eq. (D1); adding a delay node to the self-inhibition of X [Eq. (D2)] equalizes the feedback loop lengths and results in monostability under synchronous update, consistent with the results of [67]. In asynchronous update, both systems are monostable. See panel A of Fig. 10 for further details.

(D1)
$$X^{⋆}=\neg X\;\wedge \;Z,\;Z^{⋆}=X,$$

(D2)
$$X^{⋆}=\neg Y\;\wedge \;Z,\;Y^{⋆}=X,\;Z^{⋆}=X.$$

### 2. Aurora Kinase A in Neuroblastoma

The Aurora Kinase A in Neuroblastoma () model developed by Dahlhaus *et al.* [37] explores the role of the Aurora Kinase A protein in the cell cycle of neuroblastoma cancer cells. Dahlhaus *et al.* used synchronous update and reported three families of attractors: a point attractor corresponding to the G0 checkpoint, a three-state cycle describing cells proceeding faithfully through mitosis, and a three-state cycle corresponding to cells with defective mitosis, respectively. Aurora Kinase A is off in the G0 point attractor, expressed and active in the faithful mitosis attractor, and oscillates in the defective mitosis attractor. Defective mitosis leads to mitotic catastrophe and cell death via mechanisms outside the model, and is desirable in the context of neuroblastoma. Dahlhaus *et al.* find that constitutive activation of Greatwall/MASTL stabilizes Aurora Kinase A, increasing the likelihood of faithful mitosis of cancer cells and decreasing the likelihood of mitotic catastrophe. Analysis of gene expression profiles of neuroblastoma patients confirmed that constitutive activation of Greatwall/MASTL is correlated with poor prognosis.

In asynchronous update, only attractors corresponding to the G0 checkpoint and faithful mitosis exist. This also leads to population-level differences in this model: Aurora Kinase A is active significantly more often under synchronous update than under asynchronous update, yielding a higher average expression level of Aurora Kinase A in a cell population. This model can be reduced to the system

(D3)
$$\;AK^{⋆}=PLK1\;\wedge \;AKP,\;AKP^{⋆}=\neg PP2A,\;\;MP^{⋆}=\neg MP\;\wedge \;(AK\;\vee \;PLK1),\;PLK1^{⋆}=AK,\;PP2A^{⋆}=\neg AK\;\wedge \;\neg MP.$$

Here, AKP and AK represent the presence and activity of the Aurora kinase A, respectively; PP2A and PLK1 are important cell cycle proteins, and MP represents the physical processes of mitosis. As in the full model, this reduced system has synchronous-update attractors corresponding to the G0 checkpoint and faithful and defective mitosis; the last of these vanishes in asynchronous update, leading to differences in the average activity of Aurora kinase A. Notably, the synchronous behavior is sensitive to the existence of the intermediary node AKP: if AKP and AK are merged, the synchronous update yields similar results to the asynchronous update, which is insensitive to this merger. This shows that the defective mitosis attractor is dependent on a delay between PP2A activation and its effect on AK. Because delays are intrinsically stochastic in the asynchronous update, this delay dependency explains why defective mitosis cannot be sustained under asynchronous update.

We note that the main conclusion of the original article, that stabilization of the Aurora kinase increases mitosis of cancer cells, does not depend on the existence of the defective mitosis attractor.

### 3. Regulation of the L-arabinose operon in *Escherichia coli*

In contrast to the previous examples in this section, the Regulation of the L-arabinose operon in *Escherichia coli* () model [38] has spurious synchronous attractors that disappear under asynchronous update and do not have biological meaning. This model describes the regulation of the genes involved in arabinose metabolism in *E. coli* in different environmental settings. Specifically, the model considers 12 possible combinations of three levels of external arabinose, availability of unbound AraC protein, and the presence/absence of external glucose. In the input configuration corresponding to a medium level of external arabinose, available unbound AraC protein, and no external glucose, there are two point attractors, and four additional cyclic attractors under synchronous update. As in the example of Eq. (1), these additional synchronous attractors arise from a positive feedback loop (here formed by four nodes), and the symmetry of the positive feedback loop causes the average node values to be unaffected by the additional attractors. The original article describes these additional attractors as artifacts of the synchronous update, in contrast to the two biologically justified point attractors shared by both updates.

The timing dependence of the model’s attractors is only observed in this specific input configuration. The model is monostable (has a single, update-independent point attractor) in the remaining 11 input configurations.

### 4. Metabolic Interactions in the Gut Microbiome

The Metabolic Interaction in the Gut Microbiome () model [40] describes inferred interactions among 10 bacterial genera of the healthy gut microbiome, the pathogenic bacterium Clostridium difficile, and clindamycin antibiotic treatment. When clindamycin is present, the system reduces such that the attractor is determined by a complete subnetwork of three cooperative and self-sustaining bacterial genera (Lachnospiraceae, Lachnospiraceae_other, Other). As a consequence, the basin of the attractor in which all three genera are present, representing more than 85% of the state space, is identical in the two update schemes. The remaining state space is split between two very similar attractors in a manner that only weakly depends on update scheme. These effects can be seen by comparing the state transition graphs of this model under synchronous and asynchronous update (see panel B of Fig. 10). In the absence of clindamycin, only two nodes are free to vary and their average values depend mildly on update scheme.

### 5. Colitis-associated colon cancer

The Colitis-associated colon cancer () model [41] has an unusually high difference in average node values depending on which update scheme is used (see Fig. 3). This model integrates the signaling pathways that underlie inflammation-associated tumorigenesis. The original analysis used asynchronous update and reported three oscillating attractors and two point attractors, each of the latter having a very small basin of attraction. Notably, the authors also emphasize the average node values in their interpretation of the model, meaning that the large difference in average node values under the two update schemes may be especially significant. The authors also identify a core regulatory subnetwork that determines the dynamics of the system under protumor conditions. Within this subnetwork, we identify that the majority of the difference in average node values stems from the relationships between three nodes: CTL, IFNG, and IL10,

(D4)
$$\;CTL^{⋆}=IFNG\wedge \neg IL10,\;IFNG^{⋆}=CTL,\;\;IL10^{⋆}=\neg IFNG.$$

This three-node network is analyzed in panel C of Fig. 10. It has two point attractors. Under synchronous update, one of these attractors is not reachable from any other state. Under asynchronous update, however, most states can reach either attractor. Because these two attractors have all three nodes in opposite states, this gives rise to a large RMS difference in average node values, which propagates through much of the network.

### 6. Cortical Area Development

Cortical Area Development () model [42] aims to explain how interactions among a morphogen and four transcription factors lead to their characteristic expression pattern during mouse cerebral cortex development. The two poles of the cortex are represented by different initial conditions. The model uploaded to the Cell Collective was featured in [42] as a previously hypothesized model that does not recapitulate the expected biological result. We analyzed the model on the Cell Collective as well as one of the successful models reported in [42]. Both are bistable, with one attractor being much more likely than the other under synchronous update; under asynchronous update, the two attractors are more equally balanced. The state transition graph of the Cell Collective version is shown in panel D of Fig. 10. The state transition graph of the more successful model exhibits similar behavior, but the sizes of the two attractor basins are interchanged. The model in the Cell Collective is not successful under either update; the other model requires asynchronous update for success. Thus, the biological interpretation of the improved model is strongly dependent on update scheme.

### 7. Apoptosis Network

The Apoptosis Network () model [43] of Mai and Liu describes cancer cells’ decision between apoptosis and survival. Mai and Liu used synchronous update and reported that both phenotypes are possible under each combination of growth factor and tumor necrosis source nodes. We confirm this and identify a three-node subnetwork that determines the phenotype,

(D5)
$${\mathrm{Cas3}}^{⋆}=\mathrm{Cas}6\;\wedge \;\neg IAP,\;{\mathrm{Cas6}}^{⋆}=\mathrm{Cas}3\;\wedge \;\neg IAP,\;IAP^{⋆}=\neg \mathrm{Cas}3\;\vee \;\neg \mathrm{Cas}6.$$

Apoptosis occurs when Cas3=Cas6=1 and IAP=0, while Cas3=Cas6=0 and IAP=1 lead to survival. Our analysis with cubewalkers found that the outcome of both the full model and this subnetwork strongly depends on update scheme: apoptosis is twice as likely under asynchronous update (see panel E of Fig. 10). In the full model, changing the update scheme changes whether survival or apoptosis is more likely. Despite this dramatic difference, enough nodes in the network take the same value in both attractors that the network’s average node values overall are moderately robust to update scheme. Indeed, the model has an RMS difference in average node values that, though higher than the median, is quite low in absolute terms (near 0.1; see Fig. 3).

Thus we have observed that the attractors and average node values in this model are robust to timing perturbation, but the biological interpretation of the dynamics is only partly conserved across update schemes.

### 8. Human Gonadal Sex Determination

The Human Gonadal Sex Determination () model [48] describes the gene regulatory network that controls the differentiation of the gonadal primordium towards testes or ovaries in the early stages of embryonic development. The original article reported three point attractors; in addition to the two expected ones, each with a basin of almost 50% under synchronous update, there is a third attractor, corresponding to disgenetic testes, whose basin is less than 1%. We find that under asynchronous update, the basin of the two expected attractors decreases and the basin of the third attractor increases. We note that this model has high fragility $\left(\varphi_{a,f}\approx 1.1\right.$, and $\varphi_{s,f}\approx 1.5$ for synchronous and asynchronous update; the model has no source nodes). Fragile models such as this are characterized by multiple basins of attraction with attractors that differ in many nodes. When a node of the system is perturbed, the system has a tendency to enter a different basin of attraction, causing its converged average node values to be substantially different than those of the unperturbed trajectory.

A four-node reduced version of the Human Gonadal Sex Determination model () illustrates this property,

(D6)
$$CTNNB1^{⋆}=WNT4\wedge \;\neg SRY,\;\;SOX9^{⋆}=\neg WNT4\;\wedge \;SOX9\;\wedge \;\neg CTNNB1,\;\;\;SRY^{⋆}=\neg CTNNB1\wedge (SOX9\;\vee \;SRY),\;\;WNT4^{⋆}=\neg SOX9\;\wedge \;\neg SRY.$$

This reduced model has three attractors, one of which has a basin of attraction much larger than the others (11 states versus 2 and 3 states). The two attractors with smaller basins of attraction are highly fragile; a perturbation to a single node has a 75% chance of altering the attractor basin in four out of five of these states, and a 50% chance of doing so in the fifth state. Though these attractors have small basins, collectively they make up just under a third of the state space. The resulting fragility in this reduced model is 1.125 under synchronous update. We conjecture that the abundance of overlapping mutual inhibition loops in the reduced model contributes to the fragility of the attractor basins. See Fig. 11 for a detailed visualization of the fragility of this reduced system.

## APPENDIX E: SOURCE NODES AND CONSTANT NODES ARE RARE IN RBNs

Source nodes are rare in most types of RBN ensembles. To illustrate this, consider an RBN ensemble with a specified in-degree distribution, P(k), and assume that a node with a given in-degree has its regulators chosen uniformly at random. We also assume that regulatory functions are chosen as in the N−K model with bias p. In such a random model, the probability that a node with in-degree k self-regulates is k/N, for a network of N nodes. The probability that the source update function [e.g., $f_{i}(x)=x_{i}$] is chosen is $p^{2^{\left(k-1\right)}}(1-p{)}^{2^{\left(k-1\right)}}$ because for the half of the $2^{k}$ possible inputs in which $x_{i}=1$, an output of 1 must be chosen, while for the other half, 0 must be chosen. Therefore, the probability that a node with k regulators and bias p is a source node is

(E1)
$$P_{\text{source}}(k,p)=\frac{k\sigma^{2^{k}}}{N},$$

where we have used the bias variance, $\sigma^{2}=p(1-p)$, to simplify the expression.

Thus, the probability that a specific node is a source node is $\sum_{k=0}^{\infty }P_{\text{source}}(k,p)P(k)$. By assuming that node properties are generated independently, the expected number of source nodes can be calculated by multiplying by *N*:

(E2)
$$\langle n_{\text{source}}\rangle =\sum_{k=0}^{\infty }P(k)k\sigma^{2^{k}},$$

Notably, this expression is independent of N. This is because there are two competing effects as the network size grows that exactly cancel out on average: (i) with more nodes, there are more potential source nodes, and (ii) with more nodes, there are more potential regulators for each node, making it less likely that a node selects itself as a regulator.

We now put an upper bound on $\left\langle n_{\text{source}}\right\rangle$. The largest σ can be is 1/2, which is obtained for p=1/2. This allows us to write $\left\langle n_{\text{source}}\right\rangle ⩽\sum_{k=0}^{\infty }P(k)k2^{-2^{k}}$. The expression $k2^{-2^{k}}$ is maximized for k=1. Substituting this provides a numerical upper bound on the expected number of source nodes

(E3)
$$\langle n_{\text{source}}\rangle \;⩽\;1/4.$$

Because the expected number of source nodes is bounded above by 1/4, and because the number of source nodes in any finite network must be a non-negative integer, we expect that in any ensemble of finite random networks (generated according to the assumptions above), more than 75% completely lack source nodes. This stands in stark contrast to the Cell Collective; only nine of these 72 models are source-free, and the average number of source nodes in these networks is 4.94 (median 3, maximum 33) (see Fig. 12).

A similar calculation can be performed to determine the expected number of constant nodes in these models. The probability that a node with k regulators has an update function equal to 1 is $p^{2^{k}}$ (because an output must be chosen for all $2^{k}$ input configurations). Similarly, the probability that this node has the update function 0 is $(1-p{)}^{2^{k}}$. Thus, the expected number of constant nodes is

(E4)
$$\langle n_{\text{constant}}\rangle =N\sum_{k=0}^{\infty }P(k)\left(p^{2^{k}}+{\left(1-p\right)}^{2^{k}}\right).$$

For p=1or0, all nodes are constant; for p=0.5, the fraction of constant nodes is minimized and can be made arbitrarily small by weighting the in-degree distribution toward higher k.

## APPENDIX F: SUPPLEMENTARY FIGURES

Figures 13–19 present additional information regarding the distributions of measures discussed in the main text.

## APPENDIX G: SUPPLEMENTARY TABLES

### 1. Modifications to Cell Collective models

Tables II–IV present information regarding modifications we have made to models in the Cell Collective.

**TABLE II.** Modifications to account for source nodes that express a cellular context.

Model name	PMID	Modification
Bortezomib Responses in U266 Human Myeloma Cells	26163548	Constant source nodes: SHP1 = 0 and TNFA = TNFAR = X = 1.
CD4 T cell signaling	25538703	Constant source node: CAV1_ACTIVATOR = 0.
EGFR & ErbB Signaling	19662154	Constant source nodes: mkp = pp2a = pp2b = 0 and erbb1 = erbb2 = erbb3 = erbb4 = pten = ship2 = csrc = pdk1 = esp8r = mtorr = pi3kr = sos1r = 1.
Glucose Repression Signaling 2009	19144179	Constant source nodes: GAL11 = GAL2 = GAL80 = GLC7 = GRR1 = MALT = MIG1 = REG1 = RGT1 = RGT2 = SNF1 = SNF3 = SNF4 = STD1 = YCK1_2 = 1.
Guard Cell Abscisic Acid Signaling	16968132	Constant source nodes: ABH1 = ERA1 = GCR1 = 1.
HGF Signaling in Keratinocytes	22962472	Constant source nodes: AKAP12 = PTEN = DUSP1 = 0 and PAI-1 = 1.
HIV-1 interactions with T Cell Signalling Pathway	25431332	Constant source nodes: RASA = 0 and antigen = BCAR1 = CD45 = Chemokine = CRKL = DLGH1 = GADD45 = GRKL = ICOS = IKBNFKB = PDCD1_PD1 = 1.
IL-1 Signaling	21968890	Constant source nodes: irakm = pten = sil1r12 = smyd88 = socs1 = socs3 = 0 and abin2 = ck2 = ikka = ikkb = mtorc2 = pdk1 = 1.
IL-6 Signalling	21968890	Constant source nodes: cyt_ptpe = gp130m = nfkb = phlpp = pias1 = pias3 = pten = ros = ship = sirp1a = slim = 0 and gab1_kin = mtor = pdk1 = 1.
T Cell Receptor Signaling	17722974	Constant source node: lckr_input=1.
T-LGL Survival Network 2008	18852469	Constant source nodes: TAX = CD45 = 0 Misspelling fixed: IFN should be IFNG in CREB rule.
T-LGL Survival Network 2011	22102804	Constant nodes: TAX = CD45 = 0.
BT474 Breast Cell Line Long-term ErbB Network	24970389	The isolated source node BAX is removed.
HCC1954 Breast Cell Line Long-term ErbB Network	24970389	The isolated source nodes BAX, Nfkb are removed.
Septation Initiation Network	26244885	Constant source nodes: ppc89 = 1 and CK1 = etd1 = ras1 = 0.

**TABLE III.** Modifications to avoid invalid combinations of source node values.

Model Name	PMID	Modification
Stomatal Opening Model	27542373	As CO2_high=1 & CO2=0 is not a valid combination, we replaced CO2 by (CO2 || CO2_high) so that CO2_high=1 & CO2=0 is considered as CO2_high=1 & CO2=1.
Septation Initiation Network	26244885	As cdk_0, cdk_L, cdk_H represent levels of cdk and only one should be active, we removed the source node cdk_0 and replaced it in the rules by (!cdk_L && !cdk_H), we replaced cdk_L by (cdk_L && !cdk_H) so that combinations such as cdk_L=1 and cdk_H=1 are considered as cdk_H=1. We made cdc7 regulate sid2-mob1 as in the original paper.

**TABLE IV.** Modifications to remove aggregate source nodes and apply the original paper’s cellular context.

Model Name	PMID	Modification
Signaling in Macrophage Activation	18433497	Constant nodes: BAG4 = GAS2 = DNA = IRF4 = IFNGR2 = BCL3 = ProCASP10 = TICAM1 = NOS2Agene = MAP3K7IP2 = IKBKE = TRADD = CFLAR = JAK1 = EP300 = PTPN2 = BID = FAS = TLR9 = TLR7 = DAXX = SOCS1 = TLR5 = ProCASP8 = IFNGR1 = TRAF6 = CD40 = DFFA = TNFRSF17 = TBK1 = ProCASP4 = TIRAP = APAF1 = Proteasome = PRKRA = IL1R1 = MAP3K7IP1 = TLR2 = PRKCZ = CHUK = TLR3 = FAF1 = TICAM2 = PTP = IKBKB = FADD = MYD88 = PARP = TOLLIP = IRAK2 = TNFRSF10B = TNFRSF1B = LMNA = HSPA1A = ProCASP1 = IRF9 = TRAF5 = NFKB2p100 = SPI1 = SOCS3 = MAP3K7 = TYK2 = TLR6 = TRAF3 = TRAF2cytoplasm = RIPK3 = ProCASP2 = TNFRSF10A = TNFRSF1A = IRAK4 = RPS6KA5 = Ub = IKBKG = PRKCD = IRAK1 = BIRC2 = IFNAR2 = CREBBP = IFNAR1 = JAK2 = ATF2 = RELB = RIPK1 = 1. We removed the merged node External_Activator implemented in the Cell Collective version.
T Cell Receptor Signaling	17722974	We removed the merged source nodes unknown_input, unknown_input2, and unknown_input3 implemented in the Cell Collective version and fixed their targets in the states indicated in the original paper: akap79 = calpr1 = cdc42 = gap = pten = ship1 = 0 and bcl10 = card11 = ccblr = cd45 = gadd45 = lckr = malt1 = rac1r = 1.
Yeast Apoptosis	23233838	Constants nodes: DRE2_TAH18 = AIF1_MT = EMC4 = NDI1 = MCD1_MT = STM1_CYT = CDC48 = MMI1 = FVY10 = POR1_2 = SRO7 = SOD2 = SNO1 = SVF1 = MDV1= FIS1 = 1. We removed the merged node HK.

### 2. Model characteristics

Tables V–XIV present summary information for the models analyzed in this study.

**TABLE V.** Cancer models.

Model Name	PMID	Nodes	Source Nodes	Mean Regulators	$\delta_{s,p}$	$\varphi_{a,f}$
Aurora Kinase A in Neuroblastoma ()	26616283	23	4	2.0435	1.0504	0.9015
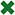
Colitis-associated colon cancer ()	26446703	70	1	2.2000	0.9867	1.5197
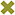
IGVH mutations in chronic lymphocytic leukemia	26088082	91	25	1.3736	0.9615	0.0014
Mammalian Cell Cycle	16873462	20	1	2.5500	0.8457	0.1941
MAPK Cancer Cell Fate Network	24250280	53	4	2.0377	1.0051	0.0105
Pro-inflammatory Tumor Microenvironment in Acute Lymphoblastic Leukemia	27594840	26	2	3.1154	0.9644	0.0034
T-LGL Survival Network 2008	18852469	60	4	3.2833	0.9202	0.1812
T-LGL Survival Network 2011 Reduced Network	22102804	18	0	2.3889	1.0125	0.3957
T-LGL Survival Network 2011	22102804	60	4	3.3167	0.8886	0.1285
Tumour Cell Invasion and Migration ()	26528548	32	2	4.9375	0.7222	0.6563
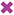

**TABLE VI.** Cancer Drug Response models.

Model Name	PMID	Nodes	Source Nodes	Mean Regulators	$\delta_{s,p}$	$\varphi_{a,f}$
Bortezomib Responses in U266 Human Myeloma Cells	26163548	67	1	1.8955	0.9147	0.2231
BT474 Breast Cell Line Long-term ErbB Network	24970389	24	5	3.0417	0.9439	0.3230
BT474 Breast Cell Line Short-term ErbB Network	24970389	16	5	3.1875	0.7614	0.1873
HCC1954 Breast Cell Line Long-term ErbB Network	24970389	23	4	3.1304	0.9643	0.4022
HCC1954 Breast Cell Line Short-term ErbB Network	24970389	16	5	3.1875	0.7841	0.1934
SKBR3 Breast Cell Line Long-term ErbB Network	24970389	25	4	3.4000	0.9458	0.2841
SKBR3 Breast Cell Line Short-term ErbB Network	24970389	16	5	2.8750	0.7908	0.3197
Treatment of Castration-Resistant Prostate Cancer	28361666	42	14	1.5476	0.9964	0.0000

**TABLE VII.** Cell Cycle models.

Model Name	PMID	Nodes	Source Nodes	Mean Regulators	$\delta_{s,p}$	$\varphi_{a,f}$
*Arabidopsis thaliana* Cell Cycle ()	26340681	14	0	4.7143	1.2722	0.0000
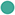
Budding Yeast Cell Cycle 2009	23049686	18	0	3.2222	1.1099	0.0003
Budding Yeast Cell Cycle	19185585	20	4	2.3000	1.0129	0.1266
Cell Cycle Transcription by Coupled CDK and Network Oscillators ()	18463633	9	0	2.1111	0.9414	0.0000
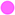
FA BRCA pathway	22267503	28	0	4.3571	1.0143	0.0036
Fanconi anemia and checkpoint recovery	26385365	15	0	4.2667	0.9783	0.0045
Mammalian Cell Cycle 2006	19118495	10	1	3.5000	1.0135	0.0000
Septation Initiation Network	26244885	30	2	1.6333	0.9014	0.2923

**TABLE VIII.** Development and Differentiation models.

Model Name	PMID	Nodes	Source Nodes	Mean Regulators	$\delta_{s,p}$	$\varphi_{a,f}$
B cell differentiation	26751566	22	5	2.0000	1.0003	0.2118
Cardiac development	23056457	15	2	2.6000	0.9866	0.1196
CD4+ T Cell Differentiation and Plasticity	30116195	18	6	4.6667	0.7214	0.4048
CD4+ T cell Differentiation	26090929	38	9	2.6316	0.9837	0.2866
Cortical Area Development ()	20862356	5	0	2.8000	0.8011	0.9384
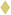
Differentiation of T lymphocytes	23743337	50	9	2.1200	0.9806	0.4199
Human Gonadal Sex Determination ()	26573569	19	0	3.9474	1.0490	1.1484
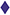
Lymphoid and myeloid cell specification and transdifferentiation	28584084	33	2	2.8788	1.0266	0.7574
Lymphopoiesis Regulatory Network	26408858	81	14	2.1235	0.9500	0.2332
PC12 Cell Differentiation	27148350	62	1	1.7581	0.9323	0.0162
T cell differentiation	6542429	23	4	1.6522	1.0314	0.6419

**TABLE IX.** Infection and Microbiome models.

Model Name	PMID	Nodes	Source Nodes	Mean Regulators	$\delta_{s,p}$	$\varphi_{a,f}$
*B. bronchiseptica* & *T. retortaeformis* coinfection	2253585	53	1	2.5660	1.0004	0.5127
*Bordetella bronchiseptica*	2253585	33	0	2.3939	1.0137	0.0453
Influenza A Virus Replication Cycle	23081726	131	11	2.3282	0.9007	0.0313
Metabolic Interactions in the Gut Microbiome ()	26102287	12	4	2.5833	1.0017	0.2718
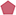
*Trichostrongylus retortaeformis*	2253585	26	1	2.2692	1.0117	0.5418

**TABLE X.** Metabolism models.

Model Name	PMID	Nodes	Source Nodes	Mean Regulators	$\delta_{s,p}$	$\varphi_{a,f}$
Cholesterol Regulatory Pathway	19025648	34	2	1.2647	0.9927	0.0000
Glucose Repression Signaling 2009	19144179	73	3	1.3699	0.7966	0.0193
Iron acquisition & oxidative stress response in *A. fumigatus*	25908096	22	2	1.8182	1.0373	0.0001
Lac Operon	21563979	13	3	1.9231	0.9974	0.0952
Regulation of the L-arabinose operon of *Escherichia coli* ()	28639170	13	4	1.6154	1.0379	0.0492
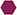
TOL Regulatory Network	23171249	24	10	2.4167	0.9347	0.0000

**TABLE XI.** Models of *Drosophila melanogaster* signaling pathways.

Model Name	PMID	Nodes	Source Nodes	Mean Regulators	$\delta_{s,p}$	$\varphi_{a,f}$
FGF pathway of Drosophila Signalling Pathways	23868318	23	9	1.3478	0.9785	0.0000
HH Pathway of Drosophila Signaling Pathways	23868318	24	13	1.8750	0.9284	0.0000
Processing of Spz Network from the Drosophila Signaling Pathway	23868318	24	6	1.4167	0.9460	0.0000
Toll Pathway of Drosophila Signaling Pathway	23868318	11	2	1.1818	1.0003	0.0000
VEGF Pathway of Drosophila Signaling Pathway	23868318	18	8	1.4444	0.9604	0.0000
Wg Pathway of Drosophila Signalling Pathways	23868318	26	14	1.6538	0.9803	0.0019

**TABLE XII.** Models of signal transduction relative to immune system cells.

Model Name	PMID	Nodes	Source Nodes	Mean Regulators	$\delta_{s,p}$	$\varphi_{a,f}$
CD4 T cell signaling	25538703	188	33	2.0160	0.9713	0.1535
HIV-1 interactions with T Cell Signaling Pathway	25431332	138	2	2.2029	0.8771	0.1888
IL-1 Signaling	21968890	118	2	1.8644	0.8375	0.0000
IL-6 Signalling	21968890	86	1	1.7442	0.7495	0.0000
Signaling in Macrophage Activation	18433497	320	18	1.4125	0.7113	0.0066
T Cell Receptor Signaling	17722974	98	3	1.5102	0.8200	0.0015
T-Cell Signaling 2006	16464248	40	3	1.3750	0.9857	0.0071

**TABLE XIII.** Models of signal transduction in stress, damage, and homeostasis.

Model Name	PMID	Nodes	Source Nodes	Mean Regulators	$\delta_{s,p}$	$\varphi_{a,f}$
Apoptosis Network ()	19422837	41	2	1.8293	1.0118	0.3297
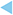
Death Receptor Signaling	20221256	28	3	1.7143	1.0350	0.8377
Guard Cell Abscisic Acid Signaling	16968132	44	1	1.7955	0.9378	0.1277
Oxidative Stress Pathway	23134720	19	1	1.7368	0.9839	0.0001
Senescence Associated Secretory Phenotype	29206223	51	2	1.9216	0.9844	0.2714
Yeast Apoptosis	23233838	72	12	1.5278	0.7210	0.0024

**TABLE XIV.** Other models of signal transduction.

Model Name	PMID	Nodes	Source Nodes	Mean Regulators	$\delta_{s,p}$	$\varphi_{a,f}$
EGFR & ErbB Signaling	19662154	104	13	2.2981	0.8128	0.0074
HGF Signaling in Keratinocytes	22962472	68	2	1.5441	0.9361	0.2023
Neurotransmitter Signaling Pathway	17010384	16	2	1.3750	0.9810	0.0234
Signal Transduction in Fibroblasts ()	18250321	139	9	3.9640	1.1178	0.3799
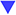
Stomatal Opening Model	27542373	49	5	3.5510	1.1772	0.0477
